# Preventive Application of House Dust Mite‐Sublingual Immunotherapy Induces Blocking Antibodies in Sensitized Preschool Children

**DOI:** 10.1111/all.70387

**Published:** 2026-05-21

**Authors:** Varsha Dwivedi, Klara Schmidthaler, Hilal Demir, Justyna Sieber, René Gaupmann, Livia Mia Gona‐Höpler, Christina Bannert, Saskia Gruber, Eleonora Dehlink, Thomas Eiwegger, Alexandra Graf, Barbara Bohle, Zsolt Szépfalusi

**Affiliations:** ^1^ Division of Paediatric Pulmonology, Allergology and Endocrinology, Department of Paediatrics and Adolescent Medicine, Comprehensive Centre of Paediatrics Medical University of Vienna Vienna Austria; ^2^ Institute of Pathophysiology and Allergy Research, Centre for Pathophysiology, Infectiology and Immunology Medical University of Vienna Vienna Austria; ^3^ Department of Paediatrics University Clinics St. Pölten Austria; ^4^ Karl Landsteiner University of Health Sciences Krems an der Donau Austria; ^5^ Translational Medicine Program Research Institute, Hospital for Sick Children Toronto Ontario Canada; ^6^ Department of Immunology, Temerty Faculty of Medicine University of Toronto Toronto Ontario Canada; ^7^ Institute of Medical Statistics, Centre for Medical Data Science Medical University of Vienna Vienna Austria

**Keywords:** allergy, blocking antibodies, house dust mite‐sensitization, preschool children, prevention, sublingual allergen immunotherapy

## Abstract

**Background:**

Sublingual allergen immunotherapy (SLIT) is an effective treatment for immunoglobulin (Ig)E‐mediated allergies. Its success is associated with allergen‐specific (s)IgG, which blocks IgE‐mediated mechanisms. Preventive effects of SLIT in children before allergy‐symptom onset remain largely unexplored.

**Methods:**

A randomized trial was conducted between October 06, 2017 and December 15, 2022, which included house dust mite (HDM)‐sensitized preschool children (aged 3–5 years) showing no allergy symptoms. They were randomized (2:2 blocks) to HDM‐SLIT (300 index of reactivity/day, Staloral) or placebo solution for 2 years. Children receiving > 4 months of treatment were included in the analysis. Primary objective of the study was to compare the groups for change in major HDM allergen‐Der p 1‐sIgG levels from baseline to end of treatment (EOT). Secondary objectives were to compare longitudinal changes (at 4 and 12 months, and EOT) in the (i) levels of HDM‐sIgG subclasses and ‐sIgE using ImmunoCAP/‐ISAC and/or ELISA (ii) Der p‐reactivity in skin and basophils using skin prick test and basophil activation test (BAT), (iii) sensitization status defined as sIgE‐positivity in skin and/or serum, and (iv) Der p‐sIgE‐blocking activity using inhibition‐BAT.

**Results:**

Eighteen children received HDM‐SLIT (mean age = 4.26 ± 0.37 years, eight females) and 15 placebo (mean age = 4.16 ± 0.59 years, nine females). HDM‐SLIT increased Der p 1‐sIgG levels between baseline and EOT versus placebo (effect size = 3.11 [95% CL 1.36–4.82], *p* = 0.001). Additional changes induced by HDM‐SLIT were: increased levels of HDM‐sIgG_1_ and ‐sIgG_4_, no long‐term increase in HDM‐sIgE levels, reduced Der p‐reactivity in skin and basophils (*p* values ≤ 0.05), and blunted development of new sensitizations. Sera from HDM‐SLIT treated individuals displayed blocking activity on basophils (*p* values ≤ 0.05).

**Conclusions:**

Preventive application of HDM‐SLIT in allergy‐prone children induces immunomodulatory effects early during treatment (elevated sIgG levels with blocking activity and reduced effector cell responses), thereby revealing potential to interfere with allergy development.

**Trial Registration:**

EudraCT#2016‐002844‐18

## Graphical abstract

HDM‐pSLIT induced IgG, IgG_1_, and IgG_4_ specific to major HDM allergens in sensitized non‐allergic preschool children without increasing HDM‐sIgE. It blunted the development of new sensitizations and reduced HDM reactivity in skin and basophils. HDM‐pSLIT treated children displayed a blocking effect on HDM‐induced basophil activation. HDM, house dust mite; Ig, immunoglobulin; pSLIT, preventively administered sublingual immunotherapy.

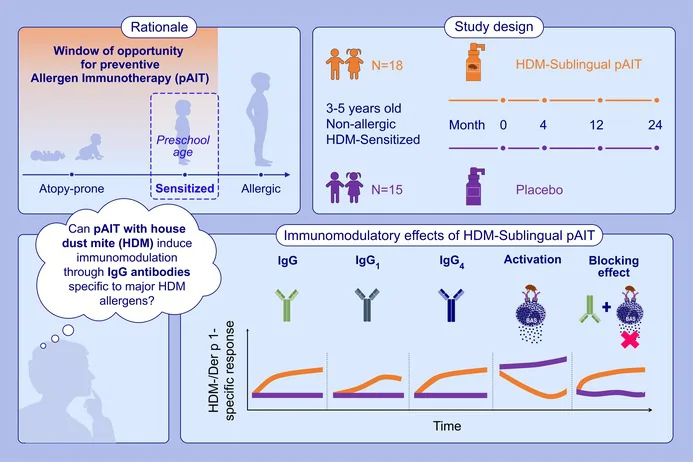

## 1 Introduction

Sublingual allergen immunotherapy (SLIT) is an effective treatment for immunoglobulin (Ig)E‐mediated allergic diseases, providing long‐term disease‐modifying benefits [1, 2, 3, 4]. Alike other routes of administering allergen immunotherapy (AIT), SLIT induces immuno‐tolerogenic effects that involve reduced frequencies, influx, and/or activation of effector cells (e.g., mast cells, eosinophils and basophils), and expanded regulatory lymphocytes (e.g., T‐ and B‐cells). By elevating regulatory cytokine levels (e.g., Interleukin (IL)‐10 and transforming growth factor‐β), SLIT induces allergen‐specific sIgG (sIgG_1_ and sIgG_4_) and sIgA antibodies that are described to block IgE‐mediated effector cell activation. Finally, sIgE levels decrease in long term [4, 5, 6, 7, 8, 9, 10, 11].

While many AIT studies focus on treating allergic diseases, a few have addressed allergy prevention using AIT [5, 12, 13, 14, 15]. Preventive AIT can be applied to different target groups: non‐sensitized non‐allergic (primary prevention), sensitized non‐allergic (secondary prevention), or allergic individuals (tertiary prevention) [2, 5, 6, 13, 15]. Delaying or preventing the natural progression of allergic disease (e.g., development of allergic rhinitis/conjunctivitis (AR/C) towards asthma) is considered the most convincing tertiary preventive effect of SLIT [2, 5, 6, 13, 15]. However, changes in sensitization within the same or to other allergen sources by SLIT remain inconsistent [5, 6, 15, 16, 17]. These effects appear most potently in young children, considering that AR/C and asthma commonly manifest at a young age [2, 4, 5, 12, 14, 18].

Secondary preventive effects of SLIT have been demonstrated with house dust mite (HDM) and grass pollen extracts in sensitized non‐allergic preschool children [19]. Although SLIT did not influence the rate of new sensitizations, it induced pro‐tolerogenic mechanisms involving IL‐10 and sIgG responses, without increasing HDM‐sIgE epitope diversity [19, 20].

The present study further explored secondary prevention with HDM‐SLIT in preschool children by investigating HDM‐specific humoral (IgE and IgG responses, particularly, the sIgG subclasses) and cellular responses (in skin and basophils). Moreover, the blocking activity of children's sera was analyzed by inhibition of HDM‐induced activation of basophils from non‐treated HDM‐allergic individuals. Additionally, the change of sensitizations by HDM‐SLIT was assessed.

## 2 Methods

### 2.1 Study Design and Participants

This was an investigator‐initiated, single‐centre, double‐blinded, placebo‐controlled randomized clinical trial (registered with European Union Clinical Trials Register; EudraCT Number: 2016‐002844‐18) conducted at the Department of Paediatrics, Medical University of Vienna, Austria. The institutional review board approved the study protocol (EK 1942/2016).

Preschool children (aged 3–5 years) were screened in the allergy outpatient ward of the study site. Included children were sensitized to HDM (either exclusively to HDM extracts or to a maximum of two additional respiratory and/or food allergen extracts). The sensitization status was defined by a positive skin prick test (SPT) (wheal size > 3 mm) [21] and/or sIgE levels ≥ 0.35kU_A_/L. Included children showed no symptoms of seasonal or perennial allergies at baseline. Children with mild dermatitis treated effectively by groups 1–2 steroids and those with virus‐induced cough/wheeze in the last 12 months were allowed in the study. Children were excluded if during screening they (i) were sensitized to > 4 respiratory or food allergens, (ii) had food allergies, moderate–severe eczema, and/or asthma [22], (iii) were under maintenance therapy with inhalative corticosteroids, fixed‐dose combinations with long‐acting bronchodilators, and/or leukotriene receptor antagonists, (iv) were receiving immunomodulatory treatments, and/or (v) were suffering from any other severe medical condition, allergic or immunological disease. Parents or legal guardians of included children provided written informed consent.

### 2.2 Randomization and Masking

HDM‐SLIT and placebo solutions (Staloral Stallergenes‐Greer, France) [23, 24] were directly supplied to the hospital pharmacy, where eligible children were assigned to receive either treatment using computerized 2:2 block randomization. The investigators received identical boxes with study participant codes from the pharmacy to ensure double‐blinding. All participants and investigators were masked to treatment allocation until the final locked data were sent to the Centre for Medical Data Science, Medical University of Vienna, at the end of the study. The hospital pharmacy then provided the unblinded patient list for statistical analysis and had no further involvement in the rest of the trial. Data were analyzed for children who had been treated for more than 4 months.

### 2.3 Outcomes

The primary objective of the study was to compare the change (Δ) in Der p 1‐sIgG levels from baseline to end of treatment (EOT) between HDM‐SLIT and placebo.

Secondary objectives involved assessing longitudinal differences between and within the treatment groups for baseline‐normalized Δ‐values of (i) Der p 1‐sIgG at other time points (4 and 12 months), (ii) sIgG, sIgG_1_, and/or sIgG_4_ levels for additional major HDM allergens (Der p 2 and 23), Der p‐extract, and other non‐treatment allergens, (iii) sIgE levels for HDM‐extracts and individual allergens, (iv) Der p‐reactivity in skin and basophils, (v) number of sensitizations to allergen‐extracts (in serum and skin, combined) and individual allergens (in serum), and (vi) IgE‐blocking activity of children's sera. All objectives were assessed at 0 months (baseline), 4 and 12 months, and EOT.

### 2.4 Procedures

Randomized children received either a daily maintenance dose of 300 index of reactivity (IR)‐HDM‐SLIT or placebo solution for 2 years, following manufacturer's recommendations (Supplementary info_Method_1). Children were regularly monitored every 2 months for treatment compliance and their caregivers were instructed to record all clinical events, vaccinations, and other medical treatments in analogue diaries. Their demographic data were recorded during medical assessments followed by blood sample collection and SPT at baseline, 4 and 12 months, and EOT.

Serum levels of sIgG and sIgG_4_ were measured using ImmunoCAP (Thermo Fisher Scientific, Denmark). Levels of sIgG_1_ for *Dermatophagoides pteronyssinuss* (Der p)‐extract (generously provided by S. Vrtala, Medical University of Vienna), natural Der p 1 (nDer p 1), and nDer p 2 (Inbio, United Kingdom) were measured using enzyme‐linked immunosorbent assay (Supplementary info_Method_2). A standard panel of 10 common respiratory and food allergen extracts, including HDMs (Der p) and *D. farinae* (Der f), was used to measure sIgE levels through ImmunoCAP (Thermo Fisher Scientific Inc.) and skin reactivity through SPT (without Der f) (ALK‐Abello, Denmark) (Supplementary info_Method_2 and 3). ImmunoCAP/‐ISAC (Thermo Fisher Scientific, Denmark) was used to measure sIgE levels for an array of 112 individual allergens (Supplementary info_Method_2).

Children's basophil responses (%CD63 positivity) to titrated Der p‐extract were measured using an in vitro basophil activation test (BAT) kit, following manufacturer's instructions (FlowCAST, BÜHLMANN Laboratories AG, Switzerland). Basophil responses were used to quantify the allergen concentration required to induce half‐maximal basophil activation (EC_50_ (ng/mL)) and area under dose–response curve (AUC) calculations representing basophil sensitivity and reactivity [25, 26] (Supplementary info_Method_4). Blocking activity of children's sera was assessed through inhibition (i) BAT, as described earlier [27, 28, 29, 30]. Briefly, Der p‐extract, nDer p 1 and nDer p 2 were pre‐incubated with children's sera before addition to the whole blood from HDM‐allergic donors, followed by BAT (Supplementary info_Method_5). HDM allergy was confirmed by clear history of perennial allergy, positive SPT, and/or elevated sIgE levels in the donors. Donors were sampled after receiving a written informed consent (EK 1344/2018).

Standard scores used to monitor existing symptoms and medication use [31] were not investigated, given the non‐allergic status of the cohort. General health of participants was monitored; the occurrence of symptoms affecting skin and airways was recorded and their intensities were graded subjectively. Adverse events (AEs) were documented at each visit by investigators and in analogue diaries by parents (Supplementary info_Method_6 and 7).

### 2.5 Statistical Methodology

Previous results on the differences in Der p 1‐sIgG levels between SLIT and placebo [19] were used for sample size calculation. These results led to an assumed effect size (ES) of 0.32 (probability that an observation in group one is less than one in group two). A sample size of 41 in each group would have 80% power to detect the assumed effect using a Wilcoxon (Mann–Whitney) rank‐sum test with a two‐sided significance level of 0.05. The primary endpoint, change of Der p 1‐sIgG levels from baseline to EOT, was compared between treatment groups using a linear mixed regression model accounting for time (all measured time points), group, the interaction between time and group as well as age, sex and family history as fixed and subject ID as random factor. Note that the change from baseline to EOT was the primary outcome of interest; the other time points (4 and 12 months) were considered as secondary outcomes. Therefore, no correction for multiplicity was performed and a *p* value < 0.05 for the primary outcome was considered as statistically significant. A negative (positive) effect, for 24 months versus baseline, indicates a larger (smaller) change in the value (at 24 months vs. baseline) for placebo as compared to HDM‐SLIT. Results from regression models are presented as ES [95% Confidence level (CL)], *p*‐value.

Secondary parameters were compared between groups in a similar way, however, for some of the secondary parameters, median instead of linear regression models, depending on the data skewness, were performed. All secondary *p* values ≤ 0.05 were considered statistically significant. *p* values and widths of the intervals for secondary parameters were not adjusted for multiplicity and results may therefore be interpreted with caution. Further, as sensitivity analyses, the models were repeated without group factor to evaluate the time‐trend within the groups (Supplementary_Info_Method_8). Responses in iBAT were analyzed using Mann–Whitney *U* test and Dunn's multiple comparisons test to assess between‐ and within‐group differences in time‐trend, respectively. Categorical variables were compared between and within groups using Fisher's exact test. All statistical evaluations and graphs were generated using R (version 4.2.2) and GraphPad Prism (version 10.4.1).

## 3 Results

Children with HDM sensitization at preschool age were screened between October 6, 2017 and December 15, 2020. Thirty‐nine children consented to participate and eligible children were randomized to HDM‐SLIT (*n* = 20) or placebo (*n* = 17). Data were analyzed from 18 and 15 children who received HDM‐SLIT or placebo for more than 4 months, respectively (Figure 1). Altogether, 16 children from the HDM‐SLIT group and 13 from the placebo group completed 2 years of treatment. Baseline demographics of both groups are listed in Table 1.

**FIGURE 1:**
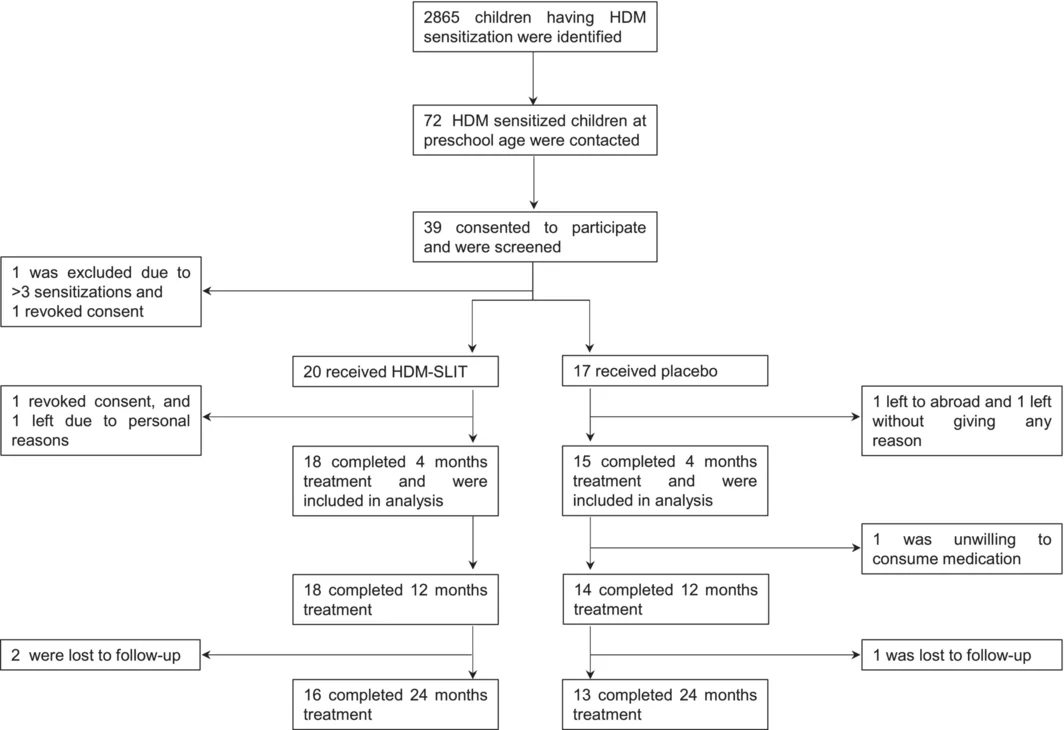
CONSORT flow chart. Flow chart showing screening, randomization and participation throughout the study. HDM (House Dust Mite), HDM‐SLIT (House Dust Mite Sublingual Immunotherapy).

**TABLE 1:** Baseline demographics.

Demographics	HDM‐SLIT (*N* = 18)	Placebo (*N* = 15)
Age (years)	4.3 ± 0.4	4.2 ± 0.6
Sex		
Male	10, 55.6%	6, 40%
Female	8, 44.4%	9, 60%
BMI (kg/m^2^)^a^	16.7 (14.6–20.9)	14.9 (12.4–18.9)
Breastfed (Yes)	15, 83.3%	13, 86.7%
Age at start of infant feeding formula (months)^a^	5 (3–8)	5 (3–10)
Paternal history of confirmed allergy	5, 27.8%	3, 20%
Maternal history of confirmed allergy	6, 33.3%	6, 40%
Patients having siblings with allergy	1, 5.5%	3, 20%
Nicotine exposure (Yes)	6, 33.3%	6, 40%
Reason for allergy testing		
Infection‐related symptoms in upper/lower airways	9, 50%	9, 60%
Doctors' referrals from other allergy centres	3, 16.6%	5, 33.3%
Positive family history	6, 33.3%	1, 6.6%

^a^ Categorical variables are presented as *n*, % and continuous variables as mean ± standard deviation or median (minimum–maximum range), depending on the data distribution.

The study was successful in its primary objective, observing a significantly larger increase in the HDM‐SLIT group than the placebo group in Der p 1‐sIgG from baseline to EOT (24 months: ES = 3.11 [95% CL 1.36–4.82], *p* = 0.001). Furthermore, the increase of Der p 1‐sIgG levels from baseline to 4 and 12 months (secondary time points) was significantly larger in the HDM‐SLIT group than in the placebo group (4 months: ES = 2.62 [0.96–4.27], *p* = 0.003; 12 months: ES = 3.60 [1.90–5.28], *p* < 0.001) (Figure 2ia–b and Table S1.a.i). Increase in Der p 1‐sIgG_1_ and ‐sIgG_4_ levels was also significantly larger in the HDM‐SLIT group than in the placebo group at each time point from baseline to EOT (all differences with *p* values < 0.05). HDM‐SLIT group presented gradual increases in the levels of additional major allergens Der p 2‐, Der p 23‐, and/or Der p ‐sIgG, ‐sIgG_1_, and ‐sIgG_4_ from baseline and EOT, which was significantly larger than the placebo group (all *p* values < 0.05), except for Der p 23‐sIgG_4_ (Figure 2ii–iv and Table S1.a‐c). Changes in sIgG and sIgG_4_ for non‐treatment allergen sources showed no significant between‐groups differences, except for *Ambrosia*‐sIgG levels (Figures S1 and S2; Table S1.d).

**FIGURE 2:**
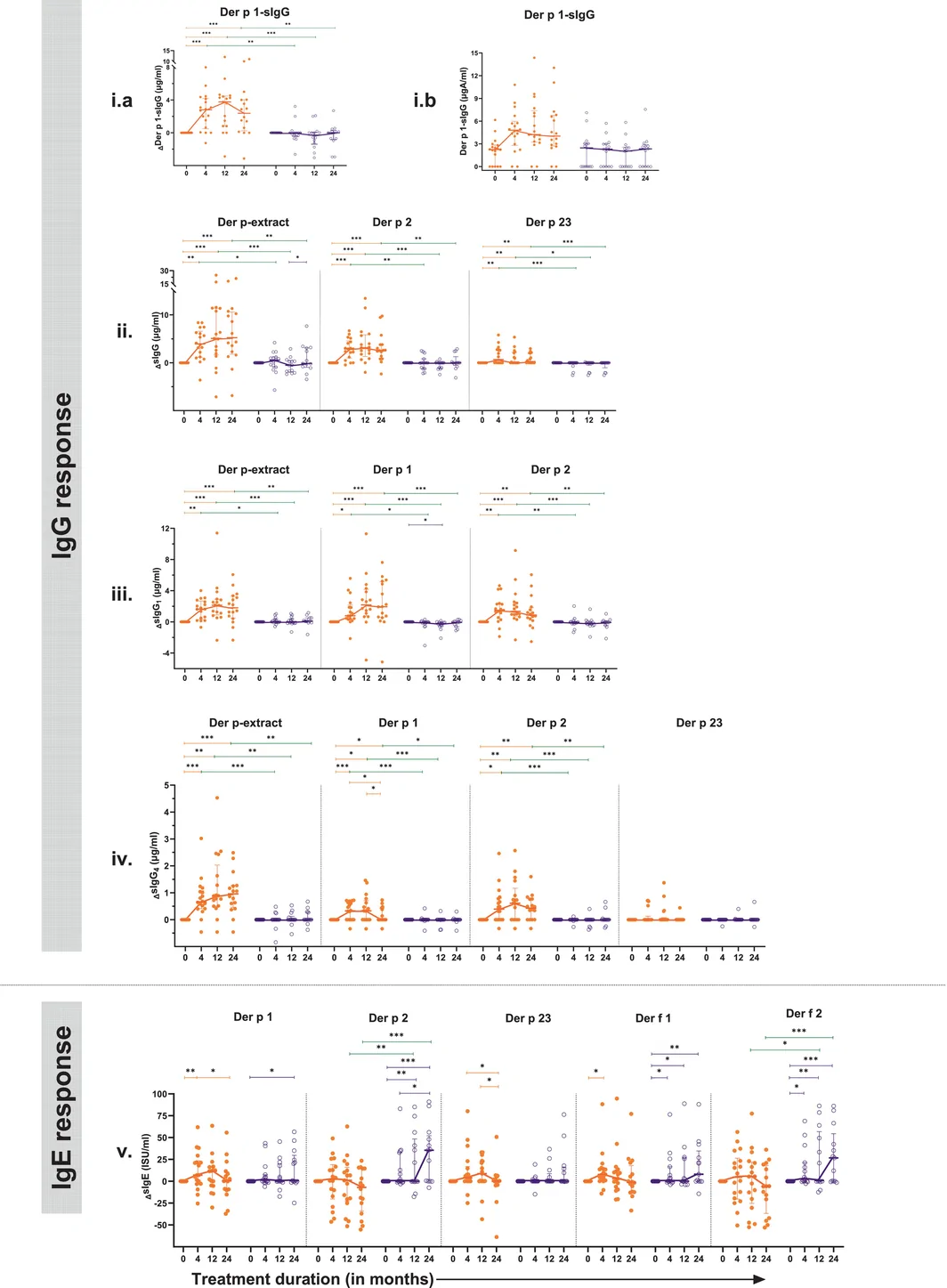
Humoral changes in sIgG, sIgG_1_, sIgG_4_, and sIgE levels for major HDM allergens and extracts induced by HDM‐SLIT. (i.a,b) Changes in Der p 1‐sIgG from baseline to EOT are shown as delta (Δ)‐values alongside absolute levels of Der p 1‐sIgG (μg/mL),^a^ (ii–iv) Baseline‐normalized Δ‐values of sIgG^a^, sIgG_1_^b^, and sIgG_4_^a^ levels for Der p extract and additional major HDM allergens are shown, and (v) Δ‐values of baseline‐normalized sIgE levels for HDM allergens^c^ are presented in HDM‐SLIT (filled orange circles) and placebo (open purple circles) groups. Levels of sIgE titres are presented as ISAC Standardized Units (ISU)/mL. (a) At 12 months, data were not available from one actively treated child due to technical reasons. (b) Due to limited sample availability, data were not available for sIgG_1_ measurements from two placebo cases, each at 12 and 24 months. (c) Data for sIgE response to allergen components was not available for one child each in actively and placebo groups at 12 months due to technical reasons. Statistically significant differences in time‐trend between‐groups, within the HDM‐SLIT group, and within the placebo group are shown as green, orange, and purple lines, respectively, on top of each graph. Data are presented as median [interquartile range (IQR)], *p ≤ 0.05, **p ≤ 0.01 and ***p ≤ 0.001.

Levels of Der p‐ and Der f‐sIgE increased transiently from baseline to 4 months in HDM‐SLIT group but declined thereafter until EOT, without significant differences. In contrast, the placebo group displayed an overall increasing trend for Der p‐sIgE and significant increase in Der f‐sIgE levels from baseline to EOT (*p* = 0.033) (Figure S3 and Table S1.e.i‐ii).

Major HDM allergens‐sIgE levels increased overall in the placebo group but not in the HDM‐SLIT group (Figure 2v). In detail, HDM‐SLIT increased the Der p 1‐ and Der f 1‐sIgE levels transiently at 4 months versus baseline (Der p 1: *p* = 0.009; Der f 1: *p* = 0.014), which reduced significantly thereafter until EOT for Der p 1 (*p* = 0.028) and trend‐wise for Der f 1‐sIgE. In the placebo group, Der p 1‐sIgE levels increased significantly at EOT versus baseline (*p* = 0.012) and at all time‐points for Der f 1‐sIgE (all *p* values ≤ 0.05). No significant between‐group differences were noted for Der p 1‐ or Der f 1‐sIgE levels (Table S1.e.iii‐iv). HDM‐SLIT reduced Der p 2‐ and Der f 2‐sIgE significantly more than the placebo (Der p 2‐ baseline vs. 12 months: ES = −25.51 [−42.57 to −8.40], *p* = 0.005; baseline vs. EOT: ES = −40.79 [−58.09 to −23.61], *p* < 0.001 and Der f 2‐ baseline vs. 12 months: ES = −24.18 [−41.87 to −6.38], *p* = 0.011; baseline vs. EOT: ES = −35.68 [−53.63 to −17.82], *p* < 0.001) (Table S1.e.v‐vi). No significant differences between groups or within the placebo group were found for Der p 23‐sIgE levels; however, HDM‐SLIT group showed decreases in this parameter from 4 months to EOT (*p* = 0.028) and from 12 months to EOT (*p* = 0.039) (Table S1.e.vii).

Skin reactivity to titrated Der p‐extract decreased in the HDM‐SLIT group starting at 4 months for undiluted extract, while the placebo group displayed this reduction only at low allergen concentrations (Figure 3i). Loss of reactivity (median wheal size ≤ 3 mm) to 1:2 diluted extract appeared at 12 months in HDM‐SLIT group, whereas the placebo group developed this effect for 1:10 diluted extract. Significant reductions in Der p‐skin reactivity were noted in favor of HDM‐SLIT versus placebo at various allergen concentrations (baseline vs. 12 months) for 1:2 diluted extract (ES −2.90, [−5.30 to −0.50], *p* = 0.023) and baseline versus 4 months for 1:10 dilution (ES −2.80 [−4.80 to −0.82], *p* = 0.009) (Table S1.f).

**FIGURE 3:**
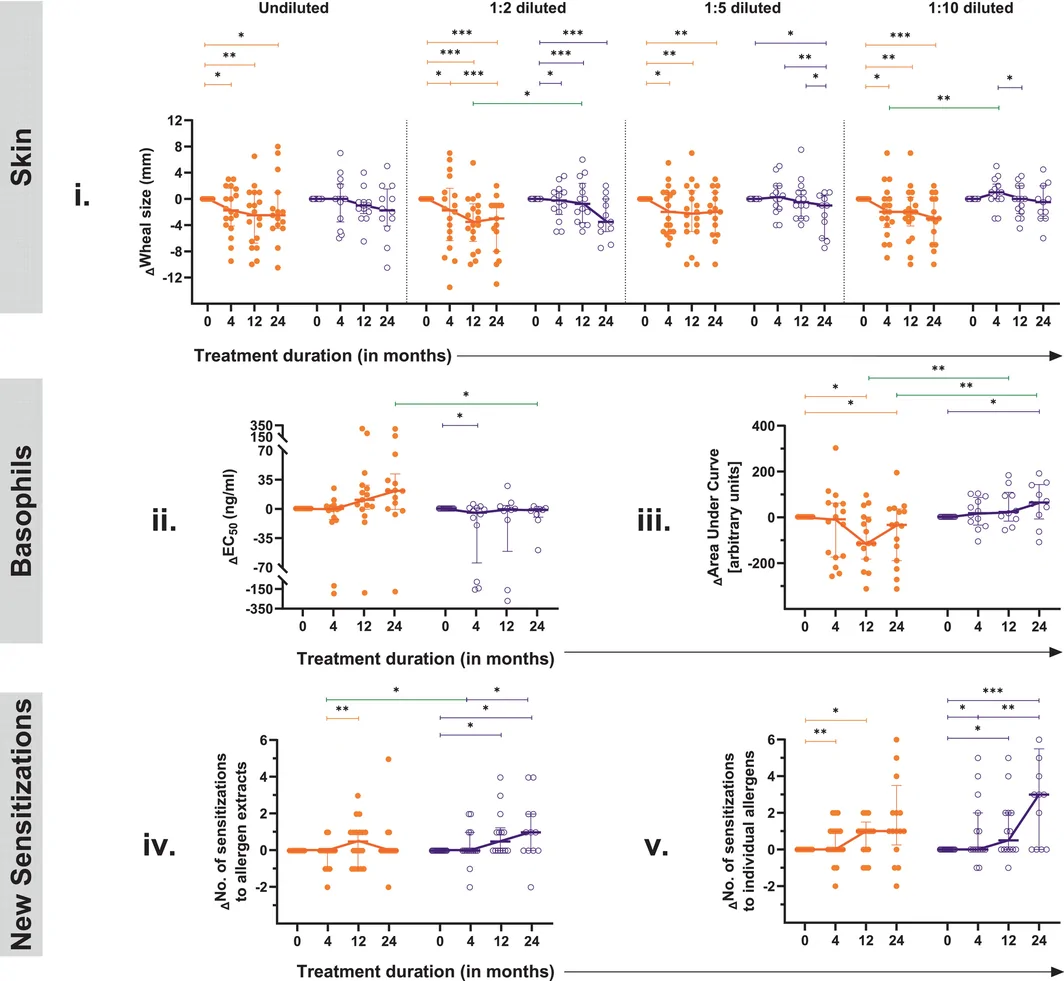
Effector cell responses to HDM‐SLIT and its impact on the occurrence of new sensitizations. (i) Baseline‐normalized Δ‐values of change in the skin reactivity to Der p‐extract (undiluted or diluted with saline as 1:2, 1:5 and 1:10) is shown. Data were not available for one placebo case at 4 and 24 months and for one actively treated case at 24 months due to technical reasons; (ii, iii) Δ‐values of baseline‐normalized basophil responses to Der p‐extract are shown as half‐maximal eliciting concentration (EC_50_) and sensitivity plus reactivity using area under curve (AUC). Data were not available from all randomized cases, and (iv, v) Δ‐values of change in number of sensitizations to 10 common allergen extracts (in skin and serum, combined) and to 112 individual components (in serum) are presented. ositive sensitization was defined as wheal diameters > 3 mm, sIgE ≥ 0.35kU_A_/L, and/or sIgE ≥ 0.3 ISU/mL. Data for sIgE to allergen components were not available for one child each in active and placebo groups at 12 months of treatment due to technical reasons. Skin sensitization status was not available for one actively treated case at 24 months and for one placebo case at 4 and 24 months of treatment. At 24 months, grass pollen‐skin reactivity data were not available for three actively treated children due to extract unavailability. HDM‐SLIT and placebo groups are indicated as filled orange and open purple circles. Statistically significant differences in time‐trend between‐groups, within the HDM‐SLIT group, and within the placebo group are shown as green, orange, and purple lines, respectively on top of each graph. Data are presented as median [IQR], *p ≤ 0.05, **p ≤ 0.01 and ***p ≤ 0.001.

Children's basophil responses to Der p‐extract were assessed in vitro (Figures S4–S6). Increase in EC_50_ (ng/mL) at EOT versus baseline for HDM‐SLIT was significantly larger than the placebo (ES = 68.86 [13.48 to 124.25], *p* = 0.016) (Figure 3ii). While EC_50_ increased trend‐wise in HDM‐SLIT group, it reduced significantly in the placebo group between 4 months and baseline (*p* = 0.036) and continued trend‐wise until EOT (Table S1.g.i). The reduction in sensitivity and reactivity (AUC) was detected for HDM‐SLIT at 12 months and EOT versus baseline and was significantly larger than the placebo (12 months: ES = −130.63 [−217.21 to −43.43], *p* = 0.005); EOT: (ES = −137.56 [−224.52 to −50.90], *p* = 0.003) (Figure 3iii and Table S1.g.ii).

Further, change in total number of sensitizations was tested in skin and sera by SPT and ImmunoCAP, respectively. A significantly stronger reduction in the total number of positive reactions was observed transiently between 4 months and baseline for HDM‐SLIT versus placebo (ES = −0.57 [−1.08 to −0.06], *p* = 0.030), which did not continue further (Figure 3iv and Table S1.h.i). In HDM‐SLIT group, the number of sensitizations increased between 4 and 12 months (*p* = 0.001) and reduced trend‐wise until EOT. In contrast, sensitizations increased significantly in the placebo group from baseline to 12 months and EOT (*p* = 0.015 and 0.013, respectively) as well as from 4 months to EOT (*p* = 0.049). At EOT, more children in HDM‐SLIT group remained mono‐ or oligosensitized (2–3 sensitizations) versus placebo group. Number of polysensitized children (having more than four sensitizations) was significantly higher in the placebo group (six children) than the HDM‐SLIT group (one child) (*p* = 0.024) (Figure S7). This indicates a hindrance in development of new sensitizations to other allergen sources in the active group.

The number of sensitizations was then analyzed independently in sera and skin (Figure S8). In sera, the number of sensitizations increased significantly between baseline and EOT exclusively for placebo (*p* = 0.049). In skin, the increase in number of sensitizations between 4 months and baseline was significantly larger in placebo group than HDM‐SLIT group (ES = −0.51 [−0.99 to −0.02], *p* = 0.040). No other significant differences were noted (Table S1.h.ii‐iii).

Additionally, a change in the number of serum sensitizations to an array of allergens was tested as a surrogate of IgE‐epitope spreading. The number of sensitizations increased significantly from baseline to EOT in the placebo group (*p* = 0.001). This was not the case for HDM‐SLIT group (*p* = 0.052) (Figure 3v and Table S1.i). Overall, sensitization to new HDM allergens occurred more frequently in the HDM‐SLIT group (*n* = 8) than the placebo group (*n* = 3). However, new sensitizations were consistently accompanied by sIgG, sIgG_1_, and/or sIgG_4_ induction exclusively in HDM‐SLIT group (Figures S9 and S10).

Thereafter, the net blocking capacity of sIgG antibodies was assessed in children's sera using iBAT (Figures S11 and S12). HDM‐SLIT‐sera from 12‐months and EOT blocked basophil activation by Der p‐extract significantly more than the corresponding placebo‐sera (both *p* values ≤ 0.05) (Figure 4i). This blocking activity emerged earlier for nDer p 1 and nDer p 2 at 4 months of treatment in HDM‐SLIT‐sera and was significantly higher than the placebo‐sera at all time‐points (all *p* values < 0.05) (Figure 4ii–iii and Table S1.j). Overall, placebo‐sera showed minimal blocking of basophil activation by Der p‐extract and nDer p 2 but significantly lowered nDer p 1‐induced basophil activation between baseline and EOT (*p* = 0.007).

**FIGURE 4:**
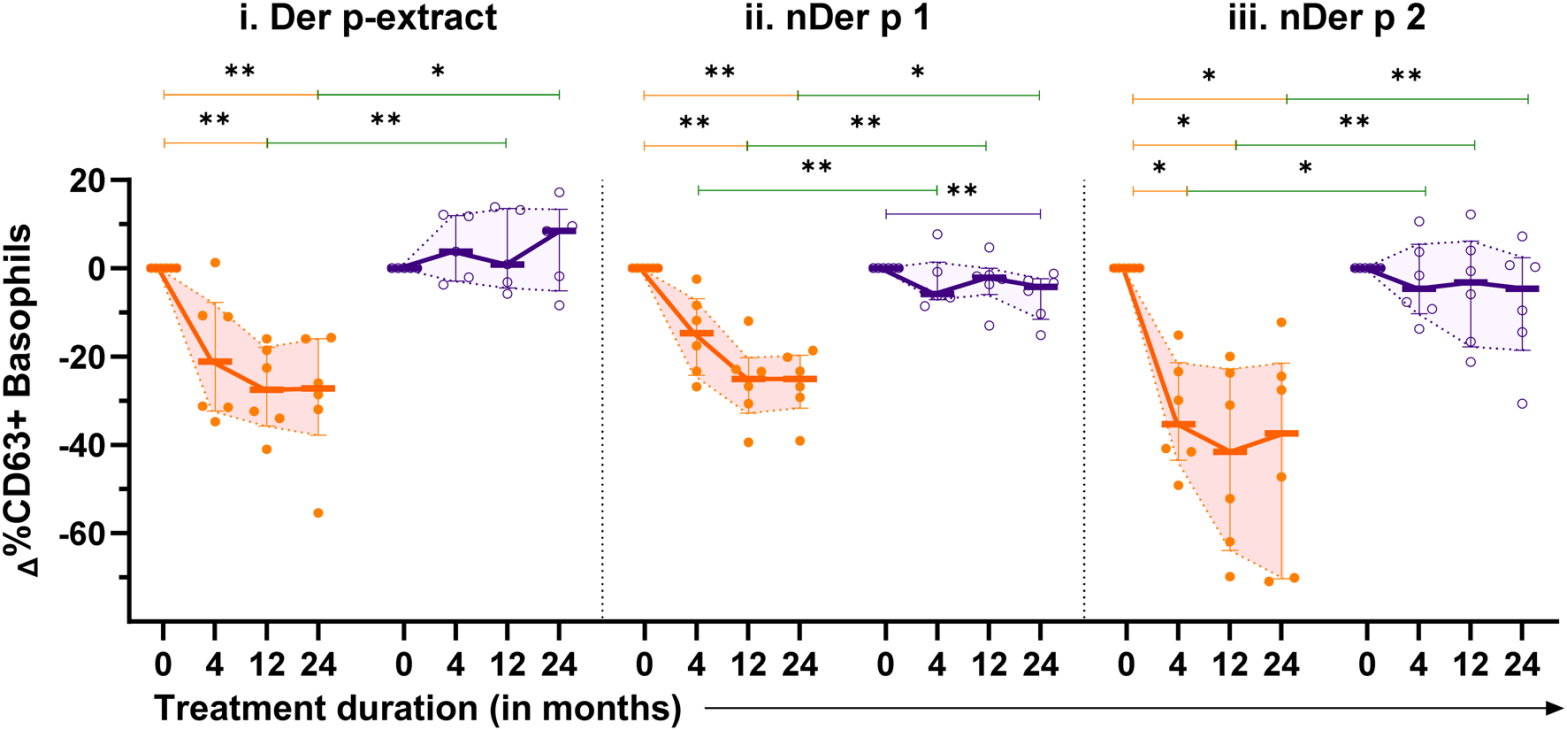
Blocking effect of children's sera on HDM‐induced basophil activation in mite‐allergic adults. Reduction in basophil activation (%CD63+ basophils) by (i) Der p‐extract, (ii) nDerp 1, or (iii) nDer p 2 incubated with sera from six children each from HDM‐SLIT (filled orange circles) and placebo groups (open purple circles) represents the blocking effect, shown as Δ‐values at various time points normalized to baseline. Basophil responses from up to four untreated HDM allergic adults were compiled to assess the blocking effect. Statistically significant differences in time‐trend between‐groups, within the HDM‐SLIT group, and within the placebo group are shown as green, orange, and purple lines, respectively on top of each graph. Data are presented as median [IQR], *p ≤ 0.05 and **p ≤ 0.01.

Lastly, the development of symptoms was assessed. Both groups displayed symptoms of dermatitis/pruritus, cough/wheeze, rhinitis, and conjunctivitis at inclusion that were not related to HDM exposure. The occurrence of these symptoms improved in both groups over the entire treatment period (Table S2). Symptom intensities were generally mild/moderate (Figure S13). AEs occurred in both groups, but none of the participants developed a serious AE possibly related to treatment. Both groups developed serious AEs unrelated/unassignable to treatment, including adenotomy, tonsillotomy, and wheezing‐bronchitis episodes requiring hospitalization. Mild to moderate AEs possibly related to treatment were recorded occasionally in both groups, including oropharyngeal itch, rhinitis, blocked nose, shortness of breath, and abdominal pain (Table S3).

## 4 Discussion

This prospective double‐blind placebo‐controlled randomized clinical trial shows that preventive application of HDM‐SLIT in sensitized non‐allergic preschool children induces IgG, IgG_1_ and IgG_4_ antibodies specific to major HDM allergens. Der p‐induced effector cell responses skin and basophils reactivity were reduced in children receiving HDM‐SLIT, indicating blocking activity of sIgG antibodies. This activity was revealed in children's sera that blocked HDM‐induced basophil activation in HDM‐allergic individuals. Beyond these specific effects, HDM‐SLIT blunted the increase in sensitizations to new allergen sources and individual allergens. These observations unanimously indicate consistent humoral and cellular immunomodulatory effects of HDM‐SLIT as an effective secondary prevention strategy for allergy development.

The preventive effect of AIT on the development of allergic asthma in AR/C‐affected children (tertiary prevention) is well recognized and supported [5, 6, 13, 16, 17, 32]. On the other hand, the preventive use of AIT is currently not recommended in healthy and/or sensitized children at risk of developing allergies (primary and secondary prevention) [15, 16]. Nevertheless, experts in the field suggest further exploring the preventive potential of AIT, particularly for secondary prevention [2, 4, 5, 6, 8, 15, 16, 33]. In accordance with a very few existing studies on secondary prevention [19, 20], the current study revealed humoral and cellular immunomodulatory effects that were also consistent with observations from tertiary prevention studies [7, 8, 32, 34, 35, 36].

So far, allergy prevention strategies using AIT have primarily focused on reducing allergy symptoms and medication use (e.g., for allergic asthma or AR/C). Since a higher number of sensitizations increases the risk of developing allergy symptoms, development of new sensitizations is often investigated, typically as a secondary outcome [13, 15, 16, 17]. Thus, the effect of AIT on new sensitizations remains inconsistent across all prevention approaches (primary, secondary, and tertiary) [13, 16, 17, 19, 33, 37]. Use of an array containing 112 individual allergens in this study revealed that HDM‐SLIT hindered the development of sensitization to other allergen sources but not to new HDM allergens. Therefore, we propose that addressing IgE‐sensitization may not be the most appropriate endpoint to assess the silencing of allergic drive towards disease manifestation. Hence, we investigated sIgG antibodies, which are considered the best biomarkers of successful AIT. AIT‐induced sIgG antibodies and their IgE‐blocking capacity have been associated with the clinical improvement of allergic diseases (such as peanut and other food allergies, AR/C, or asthma) [4, 8, 38, 39]. Our observations suggest that instead of focusing on IgE‐sensitization, tracing sIgG responses better represents the preventive properties of AIT. Ultimately, efficient induction of immuno‐tolerance by preventively applied AIT is expected to affect the onset of allergic disease [4]. However, the time frame until these clinical benefits might become relevant remains unknown and may appear beyond the active treatment phase.

Three years of therapeutic AIT are currently considered necessary for inducing long‐lasting disease‐modifying effects and have been shown to reduce the progression of grass pollen‐induced AR/C to asthma (tertiary prevention) [5, 15, 16, 32]. However, shorter durations (6–12 months) of SLIT have been tested for primary prevention in children without HDM‐sensitization [33, 37, 40, 41]. SLIT‐treated infants in these studies showed a transient reduction of new sensitizations, which was unspecific to treatment‐allergen [37]; although SLIT had no impact on the onset of allergy symptoms or immunomodulation [33, 37]. In previous secondary prevention studies, HDM‐ or grass pollen‐SLIT for 2 years induced pro‐tolerogenic effects [19, 20]. Using the same secondary prevention approach, our findings on the 2‐year treatment with HDM‐SLIT revealed early induction of immunomodulatory changes by 4 months.

Mechanistically, AIT‐induced sIgG levels (including the subclasses IgG_1_ and IgG_4_) have emerged as key humoral mediators of immuno‐tolerance [4, 6, 7, 8, 9, 11, 38, 42, 43]. Given their anti‐inflammatory characteristics, sIgG_4_ may block/suppress IgE‐mediated pathways early during treatment, reduce the infiltration, and/or activation of effector cells (e.g., mast cells and basophils) [8, 9, 27, 30, 42, 44, 45, 46]. Their blocking activity has been suggested to improve during AIT alongside the diversification of sIgG epitope recognition [20, 42]. Blocking antibodies may also reduce IgE‐dependent allergen‐uptake, maturation, and presentation of allergen by antigen‐presenting cells [9, 47, 48, 49]. Further, HDM‐SLIT boosts regulatory cells (innate lymphoid cells, dendritic cells, T‐ and B‐ cells) and cytokines that aid in re‐establishing the immune homeostasis [4, 6, 7, 8, 11]. These effects are sustained in long‐term by increased memory B‐cells that secrete blocking antibodies [4, 6, 50, 51, 52].

Our study is not without limitations. First, it did not reach the planned sample size and regression models were required to account for potential data skew. Results should therefore be handled with care. Second, it remains to be determined whether IgG_1_, IgG_4_, or both underlie the observed blocking effect, particularly in a preventive setting. Lastly, the trial continued during the COVID‐19 pandemic in compliance with the enforced hygiene regulations that reduced the overall occurrence of infectious diseases [53, 54, 55, 56], and presents a confounding factor that may have influenced the perception of respiratory symptoms in our cohort.

Nevertheless, this study is unique in reporting novel humoral and cellular immunomodulatory effects of HDM‐SLIT for secondary prevention. Having undergone the activation of primary immunity during sensitization, children in our cohort have been proposed as particularly suitable for preventive interventions [18, 33, 57]. AIT is recommended for allergic children older than 5 years [4, 5]. However, for prevention purposes, it might be initiated earlier, as shown in this study. Moreover, 300IR is a commonly used dose in therapeutic HDM‐SLIT studies [23, 58, 59, 60]. This dose was well‐tolerated in our cohort as no serious treatment‐related AEs occurred, thereby displaying potential for a new indication, i.e., preventive application. To our best knowledge, sIgG subclasses and allergen‐specific basophil responses were investigated for the first time in secondary prevention approach. Furthermore, use of a state‐of‐the‐art iBAT assay revealed first evidence that preventive application of HDM‐SLIT induces IgE‐blocking activity.

## 5 Conclusion

Preventive application of HDM‐SLIT in sensitized non‐allergic preschool children induces immunomodulatory effects (including elevated sIgG levels with blocking activity and reduced effector cell responses) early at 4 months of treatment, thereby demonstrating potential to interfere with allergy development.

## Author Contributions

V.D. was involved in conducting experiments, data analysis and curation, wrote the first draft, reviewed, edited and finalized the submitted manuscript. K.S. was involved in organizing patient follow‐up, monitoring clinical data recording, conducting experiments, data curation, reviewing and editing the manuscript. H.D. designed, performed and curated the quantification of HDM‐sIgG_1_ levels, reviewed and edited the manuscript. J.S., R.G., and L.M.G.‐H. were involved in conducting medical examinations during patient follow‐ups, recording medical data, sample collection, reviewing and editing the manuscript. C.B. and T.E. were involved in conceptualizing the study, reviewing and editing the manuscript. A.G. was involved in unlocking the blinded data lock, data exporting and cleaning, statistical analysis of data, reviewing and editing the manuscript. E.D. and S.G. were involved in reviewing and editing the manuscript. B.B. was involved in conceptualizing, designing and supervising the experiments on iBAT and sIgG_1_ measurements, obtaining ethical approval for recruiting allergic donors, funding acquisition, data analysis, editing and reviewing the manuscript. Z.S. conceptualized the study, obtained ethical approval for the study, registered the trial on the public clinical registry, acquired funding, supervised the entire study, conducted medical examination and recorded patient data, organized the patient follow‐ups, data curation, data analysis, wrote the first draft, reviewed, edited and finalized the submitted manuscript. All authors had full access to all data presented in the study and had final responsibility for the decision to submit for publication.

## Funding

This work was supported by the Austrian Science Fund, F4615‐B19, P32953, I4437. Gesellschaft für Forschungsförderung Niederösterreich, Danube Allergy Research Cluster 1.0, P12.

## Conflicts of Interests

The authors declare no conflicts of interest.

## Supporting information


**Figure S1:** Effect of HDM‐SLIT on sIgG and sIgG_4_ to non‐treatment allergens.
**Figure S2:** Effect of HDM‐SLIT on sIgG and sIgG_4_ to other non‐treatment allergens.
**Figure S3:** Effect of HDM‐SLIT on sIgE to HDM species.
**Figure S4:** Sampling flow chart for Basophil activation test.
**Figure S5:** Reference flow cytometry plots for basophil activation test.
**Figure S6:** Reference dose–response curves for BAT with Der p‐extract.
**Figure S7:** Sensitization status in HDM‐SLIT and placebo group subjects.
**Figure S8:** Number of sensitizations in serum and skin analyzed separately.
**Figure S9:** Molecular spreading.
**Figure S10:** Occurrence of new sensitizations and their corresponding dynamics with sIgG, sIgG_1_ and sIgG_4_ induction.
**Figure S11:** Dose response curves with HDM allergens for individuals with mite allergy for iBAT experiments.
**Figure S12:** Representative flow cytometry plots of iBAT.
**Figure S13:** Combined symptom scores.
**Table S1:** Detailed statistical data.
**Table S2:** Occurrence of various symptoms.
**Table S3:** Adverse events.
**Methods**. Details of treatment, measurement of analytes, data recording for the occurrence of symptoms, adverse events, and statistical analyses.

## Data Availability

Study protocol is available publicly at https://www.clinicaltrialsregister.eu/ctr‐search/trial/2016‐002844‐18/AT. Data collected during the study will be made available upon reasonable request from the corresponding author via email. Data will be shared as deidentified participant data, including informed consent forms, a data dictionary, and a statistical analysis plan. Data may be shared after signing a data access agreement.
