# Supplementary material for: Preventive Application of House Dust Mite‐Sublingual Immunotherapy Induces Blocking Antibodies in Sensitized Preschool Children
Source: Allergy. 2026 May 21;81(11):4039–50. doi: 10.1111/all.70387 (PMC13753692; doi:10.1111/all.70387)
Supplement: Supplementary file 1 [file ALL-81-4039-s001.docx]

**Supplement to:** V. Dwivedi et al. Preventive application of House Dust Mite-Sublingual Immunotherapy Induces Blocking Antibodies in Sensitized Preschool Children.

**SuppInfo_Methods.** Details of treatment, measurement of analytes, data recording for the occurrence of symptoms, adverse events, and statistical analyses.

**SuppInfo_Figure 1.** Effect of HDM-SLIT on sIgG and sIgG_4_ to non-treatment allergens.

**SuppInfo_Figure 2.** Effect of HDM-SLIT on sIgG and sIgG_4_ to other non-treatment allergens.

**SuppInfo_Figure 3.** Effect of HDM-SLIT on sIgE to HDM species.

**SuppInfo_Figure 4.** Sampling flow chart for Basophil activation test.

**SuppInfo_Figure 5.** Reference flow cytometry plots for basophil activation test.

**SuppInfo_Figure 6.** Reference dose-response curves for BAT with Der p-extract.

**SuppInfo_Figure 7.** Sensitization status in HDM-SLIT and placebo group subjects.

**SuppInfo_Figure 8.** Number of sensitizations in serum and skin analyzed separately.

**SuppInfo_Figure 9.** Molecular spreading.

**SuppInfo_Figure 10.** Occurrence of new sensitizations and their corresponding dynamics with sIgG, sIgG_1_ and sIgG_4_ induction.

**SuppInfo_Figure 11.** Dose response curves with HDM allergens for individuals with mite allergy for iBAT experiments.

**SuppInfo_Figure 12.** Representative flow cytometry plots of iBAT.

**SuppInfo_Figure 13.** Combined symptom scores.

**SuppInfo_Table 1.** Detailed statistical data.

**SuppInfo_Table 2.** Occurrence of various symptoms.

**SuppInfo_Table 3.** Adverse events.

**SuppInfo_Methods**

1. **Treatment**: HDM-SLIT (Staloral®) solution contained *Dermatophagoides pteronyssinus* and *D. farinae* (1:1) extracts. The average allergen composition of 300 index of reactivity (IR) HDM-SLIT (Staloral®) have been described as: 17.2µg/ml Der p 1, 8µg/ml Der p 2 + Der f 2, 50.1µg/ml Der f 1, and 1.64µg/ml Der p 23.^24^ A short conventional buildup scheme was followed as per the manufacturer's recommendations (Stallergenes-Greer, France). Starting from one actuation (200 µl) corresponding to 60 IR, the dose was gradually (daily) escalated to the maintenance dose of 5 actuations (1 ml) or 300 IR over 5 days. Thereafter, this maintenance dose was administered once daily for 2 years. Sublingual administration was demonstrated to the parents at randomization for a proper at-home use and treatment intake was monitored regularly by measuring the volume of interventional-products brought back to the investigators every 2 months.
2. **Levels of sIgG, sIgG_1_, sIgG_4_, and sIgE:** Serum samples were tested for sIgG and sIgG_4_ using ImmunoCAP™ (Thermo Fisher Scientific) (ranging from 2-200 mgA/l for sIgG and from 0.3- 30 mgA/l for sIgG_4_) for 18 allergens: Der p-extract, Der p 1, 2, 10, 23, cat- and dog- dander, peanut, hazelnut, walnut, grass mix, *Aspergillus*, *Alternaria*, plane tree, birch, olive, ragweed (*Ambrosia*), and mugwort (*Artemisia*).

The levels of sIgG_1_ were measured in sera (diluted 1:1 with Phosphate-buffered saline (PBS)) using an in-house enzyme-linked immunosorbent assay (ELISA) for 17 HDM-SLIT and 12 placebo-treated children. Briefly, 100µl/well of saturating concentrations of Der p-extract (25 µg/ml) (7.4mg/ml stock generously provided by S. Vrtala, Medical University of Vienna), natural Der p 1 (1 µg/ml), and nDer p 2 (1 µg/ml) (Indoor Bioscience, United Kingdom) were added to detection wells of 96-well flat-bottom microtitre plates followed by overnight incubation at 4°C in carbonate buffer (pH= 9.6). Plates were then washed twice with 250 µL/well of PBS+Tween20 (PBS-T) and blocked with 120 µL/well of PBS-T (1%) human serum albumin (HSA) for 6 hours (h) at room temperature (RT). A human Bet v 1–specific IgG_1_ monoclonal antibody was plated in duplicates to the standard wells.^28,29^ The detection range of sIgG_1_-ELISAs assays ranged from 0.195-100 ng/ml for Der p-extract and from 0.8-100 ng/ml for nDer p 1 and 2. Sera (serially diluted 1:10-1:90 with PBS-T/ (0.5%) HSA) were then added to the plate (100 µL/well). After overnight incubation at 4°C, wells were emptied and washed 5 times with PBS-T. Unlabeled mouse anti-human IgG_1_ (Invitrogen, #MA1-34581) primary antibody (diluted 1:1000 in PBS-T/ (0.5%) HSA) was added (100 µL/well) and plates were incubated at 37°C for 1h followed by another incubation at 4°C for 1h. Bound Der p-extract sIgG_1_ was detected by adding (100 µL/well) horseradish peroxidase conjugated-sheep anti-mouse IgG_1_- (enhanced chemiluminescence) (Cytiva, #NA93IV) (diluted 1:500 in PBS-T/ 0.5% HSA) following the same incubation steps as the primary antibody. For nDer p 1 and 2-sIgG_1_, rabbit anti-mouse IgG (Fc)-peroxidase (JIR, #315-035-008) (diluted 1:1000 with PBS-T/0.5% HSA) was used as detection antibody. The plates were washed 5 times after incubation as described for earlier steps. Then, 2,2'-Azinobis [3-ethylbenzothiazoline-6-sulfonic acid]-diammonium salt (ABTS) substrate was added (100 µL/well), which was composed of 10 mL citrate buffer, 10 mg of ABTS and 1 µL H_2_O_2_. Plates were stored in the dark until the measurement of absorbance at 405nm using 492nm as reference filter on a Tecan infinite F50 microplate reader (Tecan). Stored sera from all time points were tested simultaneously.

Serum levels of sIgE were tested using ImmunoCAP™ (Thermo Fisher Scientific) (cut-off= 0.35kU_A_/l) for HDM extracts (Der p and Der f), grass, birch, cat- and dog-dander, *Cladosporium, Alternaria*, peanut, hen’s egg and cow’s milk. Further, individual allergens-sIgE were measured in sera using ImmunoCAP™-ISAC™ (Thermo Fisher Scientific) (cut-off= 0.3 ISAC™ Standardized Units (ISU)/ml) that covers 112 components of 51 respiratory and food allergen sources.

3. **Skin prick test (SPT):** SPT was performed at each visit with a panel of 10 commercially available allergen extracts (ALK-Abello). Serial dilutions of Der p-extract (undiluted and diluted with saline as 1:2, 1:5, and 1:10), 6-grass-pollen mix, birch pollen, *Cladosporium herbarum, Alternaria alternata*, peanut, cat- and dog dander, hen’s egg and cow’s milk. Histamine dihydrochloride (10 mg/ml) and saline served as positive and negative controls, respectively. Single-head lancets were used. Skin reaction was recorded after 20 minutes. A wheal size more than 3 mm was considered positive for SPT. The wheal size was calculated as the average of longest wheal diameter and its perpendicular.^21^
4. **Basophil responses:** Fresh ethylenediaminetetraacetic acid- whole blood was collected from the randomized children for Basophil Activation Test (BAT) (FlowCAST®, BÜHLMANN Laboratories AG). A cocktail was prepared in 5ml centrifuge tubes with 50 µL of blood, 50 µL of Der p-extract (7.4mg/ml) at respective concentrations (1µg/ml-1pg/ml), 10 µL of staining solution, and 110 µL of stimulation buffer containing interleukin-3. This cocktail was incubated at 37°C for 25 minutes. Red blood cells were removed by adding 1 ml of lysis solution for 7 minutes. The tubes were centrifuged at 500g for 5 minutes. The supernatant was removed and wash buffer (300 µL) was added, followed by resuspension of the cell pellet. Cells were immediately acquired for flow cytometry using FACS Canto II (BD Biosciences). Data were analysed with BD FACS DIVA software (version 7.1) and FlowJo (version 10.10.0) (BD Biosciences). Stimulation buffer served as negative control (background basophil activation/ degranulation), anti-FcɛRI monoclonal antibody and N-Formylmethionine-leucyl-phenylalanine (fMLP) were the positive controls. Background degranulation was set between 2-2.5% of activated basophils (SSC^low^CCR3^+^CD63^+^) and the assay was considered valid if one of the positive controls showed ≥10% activated basophils.
   Dose-response curves were plotted to calculate the half-maximal eliciting concentration (EC_50_) (ng/ml) using [Agonist]- vs. response- variable slope (four-parameter fit) through non-linear regression fitting model in GraphPad Prism (version 10.4.1). EC_50_ (ng/ml) was calculated manually for cases not showing a well-fitted dose-response curve. Area under dose-response curve (AUC) was calculated using an in-built function in GraphPad Prism. Sensitivity and reactivity were directly proportional to AUC.
5. **Inhibition (i)BAT assays:** In these experiments, six adults allergic to HDM (not receiving maintenance therapy for symptoms) were screened for their response in BAT. Their whole blood samples were collected in lithium-heparin vacutainers and dose-response dynamics were determined for titrated Der p-extract, nDer p 1 and nDer p 2 (1µg/ml-1pg/ml) using the above-mentioned BAT kit (Supplementary figure 12). Four donors showed high reactivity (80-90% basophil activation) to extract, nDer p 1 and/or nDer p 2, who were selected for the further testing. Dose-response curves were used to estimate the optimal concentrations of Der p-extract, nDer p 1, and nDer p 2 (i.e. eliciting >30% basophil activation with baseline sera from randomised children) in presence of children’s sera. A subset of randomised children was selected for iBAT according to positivity and/or high levels of Der p-sIgG and -sIgG_4_. For iBAT, baseline sera (diluted 1:1 with PBS) from each randomised child were incubated with respective allergens at estimated concentrations for 1 hour at 37°C and basophil activation was measured to determine the optimal allergen concentration in presence of sera. Children’s sera from all mentioned time points were then stimulated similarly with corresponding optimal allergen concentrations. These allergen-sera mixtures were then added to whole blood from donors and basophil activation was measured using the BAT kit, as described above. Fixation protocol was followed after the final step of adding wash buffer, wherein the cells were incubated at RT for 30 minutes. Fixed cells were stored at 4°C until acquisition for flow cytometry.
6. **Combined symptom scores**

Occurrence of symptoms pertaining to dermatitis/pruritus, rhinitis, conjunctivitis, and coughing/wheezing was recorded by the investigators at each visit and at home by parents. The symptom intensities were graded subjectively according to participants’ and/or their parents’ (or legal guardians’) responses. The grading scales ranged from 0-10 in an ascending order of intensity for each symptom type. Symptoms were graded separately, and their average was compared between the groups as combined symptom scores ranging from 0-50.

7. **Adverse events (AEs)**

AEs were documented throughout the study as spontaneous reports from the parents/guardians in the diaries provided and/or reported during the visit to the pediatrician. Data on AEs and serious AEs were recorded in accordance with the International Conference on Harmonization (ICH) and Good Clinical Practice (GCP) guidelines.

The severity of clinical AEs was graded on a three-point scale:

- Mild- Event may be noticeable to subject; does not influence daily activities; the AE resolves spontaneously or may require minimal therapeutic intervention;
- Moderate- Event may make subject uncomfortable; performance of daily activities may be influenced; intervention may be needed; the AE produces no sequelae.
- Severe- Event may cause noticeable discomfort; usually interferes with daily activities; the subject may not be able to continue in the study; the AE produces sequelae, which require prolonged therapeutic intervention.

Note: A mild, moderate or severe AE may or may not be serious.

For all AEs, the Investigators assessed the causal relationship between the study drug and the AE using his/her clinical expertise and judgment.

Further, various types of AEs were categorized as follows:

- Gastrointestinal (GI) symptoms (e.g. nausea, emesis, diarrhea, GI- infections or abdominal pain);
- Upper airway symptoms including rhinitis (rhinorrhea and nasal congestion), epistaxis, pharyngitis, otitis, tonsillitis or post-otic tympanic effusion;
- Lower airway symptoms including cough, bronchitis or shortness of breath;
- Skin manifestations including urticaria, rash and pruritus;
- Febrile episodes;
- Planned surgical interventions (e.g. for tonsil reduction or adenotomy);
- Common paediatric infectious diseases, including viral and bacterial infection (e.g. hand-foot-mouth disease, impetigo, scarlet fever, *Varicella*);
- Uncategorized events including lymphadenopathy, palpitation, oral itching, purulent thumb infection, aphthous ulcer, headache, local insect bite reaction, helminthiasis.
  8. **Presentation of statistical analyses:**

The statistical analyses for each investigated parameter are presented in three tables. The first table shows descriptive statistics (mean, standard deviation, median, first and third quartile, minimum, maximum, and the number of valid values) over time (baseline (BL), 4-, 12-, and 24-months i.e., end of treatment (EOT)) for the two randomized groups. The results of the statistical models are given in second and third tables- one for the “Regression model for time trend” as well as one for the “Regression model for the difference randomized in time trend between groups”.

The parameters are listed in order of their appearance in the main text.

**SuppInfo_Figures**

**SuppInfo_Figure 1: Effect of HDM-SLIT on sIgG and sIgG_4_ responses to non-treatment allergens.**

Change in levels of sIgG (upper panel) and sIgG_4_ (bottom panel) are shown for grass and tree pollen allergen extracts in HDM-SLIT treated group (filled orange circles) and placebo-treated group (open purple circles) as delta (Δ)-values. Note: At 12 months follow-up, data were not available from one actively treated child in active group due to technical reasons. Linear regression model was used to compare the differences in time trend between groups. Statistically significant differences in time-trend between-groups and within the placebo group are shown as green and purple lines, respectively on top of each graph. Data are presented as median [Interquartile range (IQR)], *-p ≤ 0.05, **p ≤ 0.01 and *** p ≤ 0.001.

**SuppInfo_Figure 2: Effect of HDM-SLIT on sIgG and sIgG_4_ responses to other non-treatment allergens.**

Change in levels of sIgG (upper panel) and sIgG_4_ (bottom panel) to pet allergens (cat- and dog dander), food allergens (walnut, hazelnut and peanut), or molds (*Alternaria* and *Aspergillus*) are shown for HDM-SLIT treated group (filled orange circles) and placebo-treated group (open purple circles) as Δ-values. Note: At 12 months follow-up, data were not available from one actively treated child due to technical reasons. Linear or median regression model was used to compare differences in time trend between groups. Statistically significant differences in time-trend within the HDM-SLIT group and within the placebo group are shown as orange and purple lines, respectively on top of each graph. Data are presented as median [IQR], *-p ≤ 0.05, **p ≤ 0.01 and *** p ≤ 0.001.

**SuppInfo_Figure 3: Effect of HDM-SLIT on levels of sIgE for HDM species.**

Change in levels of sIgE for two HDM species- Der p and Der f- are shown in HDM-SLIT group (filled orange circles) and in placebo group (open purple circles) as Δ-values. Note: At 12 months follow-up, data were not available from one actively treated child due to technical reasons. Linear or median regression model was used to compare differences in time trend between groups. Statistically significant differences in time-trend within the placebo group are shown as purple lines on top of each graph. Data are presented as median [IQR], *-p ≤ 0.05, **p ≤ 0.01 and *** p ≤ 0.001.

**SuppInfo_Figure 4: Sampling flow-chart for Basophil activation test.**

**SuppInfo_Figure 5: Reference flow cytometry plots for basophil activation test.**

After removing erythrocytes, whole blood cells were resolved based on forward scatter (FSC) and side scatter (SSC). From these, the cells staining positively for chemokine cell receptor (CCR)3- phycoerytherin (PE) were gated and basophils were identified as SSC^LOW^CCR3^POS^. Finally, the surface expression of CD63-fluorescein isothiocyanate (FITC) was measured and basophil activation was recorded as %CD63+ basophils.

**SuppInfo_Figure 6: Reference dose-response plots for basophil activation test with Der p-extract.**

|  | **HDM-SLIT** | | **Placebo** | |
| --- | --- | --- | --- | --- |
| **Treatment duration** | **EC_50_ (ng/ml)** | **AUC**  **(Arbitrary units)** | **EC_50_ (ng/ml)** | **AUC**  **(Arbitrary units)** |
| **0 month (●)** | 0.93 | 86286 | 1.71 | 86868 |
| **4 months ( )** | 5.22 | 43153 | 2.48 | 87014 |
| **12 months ( )** | 17.50 | 63358 | 1.55 | 86446 |
| **24 months ( )** | 34.59 | 73163 | 0.17 | 89162 |

Data from BAT (%CD63+ basophils) were plotted for serial dilutions of Der p-extract (1µg/ml-1pg/ml) to generate a dose-response curve. These curves were then used to calculate half-maximal eliciting concentration (EC)_50_ and area under curve (AUC). Representative data from both of these parameters are shown for one HDM-SLIT treated (left) and placebo treated (right) case each at different time points (0 month- circle and dotted area; 4 months- squares and vertical-lined area; 12 months- triangles with slanted-lined area; and 24 months- diamonds and plain area).

**SuppInfo_Figure 7: Sensitization status in HDM-SLIT (upper panel) and placebo group (lower panel) subjects.**

Sensitization was determined by positivity in skin prick test (wheal size ≥3mm) and for sIgE in serum (≥0.35kU_A_/l). Children sensitized to one, 2-3, or ≥4 allergen sources were classified as monosensitized, oligosensitized, or polysensitized (black, white and red bars, respectively). Data were not available from one placebo subject at 4 and 12 months of treatment and from one actively treated subject at 24 months. At 24 months, SPT with grass pollen extract was not performed for three actively treated children due to extract unavailability. Data are presented as number of subjects in each category. Asterisk (*) indicates statistically significant differences between the groups (p= 0.0237) at the respective time point; Fisher’s exact test.

**SuppInfo_Figure 8: Number of sensitizations in serum and skin analyzed independently.**

**A.**

**B.**

A. Number of sensitizations in serum measured as positive sIgE and B. Number of sensitizations in skin measured as positive SPT. Sensitizations for various allergen sources were compared within and between HDM-SLIT (filled orange circles) and placebo-treated (open purple circles) children. Note: Data were not available for one placebo case at 4 and 24 months and for one actively treated case at 24 months of treatment because of technical reasons. At 24 months, SPT with grass pollen extract was not performed for three actively treated children due to extract unavailability. Statistically significant differences in time-trend between-groups and within the placebo group are shown as green and purple lines, respectively on top of each graph. Data are presented as median [IQR], *-p ≤ 0.05, **p ≤ 0.01 and *** p ≤ 0.001.

**SuppInfo_Figure 9: Expansion of sensitization to new HDM allergens.**

Patterns of developing a new sensitization and changes for existing sensitization to major mite allergens (Der p 1, 2, 23, Der f 1 and 2) and a minor mite allergen (Der p 10) are shown throughout the treatment in HDM-SLIT (top panel) and placebo group (bottom panel). The numbers on top represent individual subjects and those at bottom represent the treatment duration i.e. 0, 4, 12, and 24 months of treatment. Black rectangles highlight the occurrence of a new sensitization to the respective allergen component (>0.3 ISAC™ Standard Units (ISU)/ml). Levels of sIgE are shown according to the scale on the right. Note: Data for sIgE response to allergen components were not available for one child each in actively and placebo groups at 12 months of treatment due to technical reasons.

**SuppInfo_Figure 10: Occurrence of new sensitizations and their corresponding dynamics with sIgG, sIgG_1_ and sIgG_4_ induction.**

Trends in levels of sIgG, sIgG_4_ and sIgG_1_ (top to bottom) are shown with corresponding sIgE trends in HDM-SLIT group (left) and placebo group participants (right) who developed new IgE sensitizations (indicated as numbers). Note: Data on sIgG_1_ were not available for one placebo case at 12 months of treatment, marked as not-done (n.d.).

**SuppInfo_Figure 11: Dose response curves with HDM allergens for individuals with mite allergy for iBAT experiments.**

Whole blood from four HDM-allergic adults was stimulated with Der p-extract (pink dots), nDer p 1 (green dots) and nDer p 2 (blue dots) (1µg/ml-1pg/ml) to confirm reactivity in BAT and to estimate optimal concentrations for iBAT (≥30% basophil activation). Positive controls were the monoclonal antibody specific to the high-affinity IgE receptor (α-FcɛRI) (black dots) and N-Formylmethionyl-leucyl-phenylalanine (fMLP) (gray dots).

**SuppInfo_Figure 12. Representative flow cytometry plots of iBAT with Der p-extract**.

i. Control: Background

ii. Control: PC1- Anti-FCɛRI

iii. Control: PC2- N-Formylmethionyl-leucyl-phenylalanine (fMLP)

iv. Placebo_0 month

v. Placebo_4 months

vi. Placebo_12 months

vii. Placebo_24 months

viii. HDM-SLIT_0 months

ix. HDM_SLIT_4 months

x. HDM_SLIT_12 months

xi. HDM_SLIT-24 months

**SuppInfo_Figure 13. Combined symptom scores.**

Intensities of symptoms in HDM-SLIT and placebo groups (filled orange and open purple circles, respectively) are shown for dermatitis/pruritus, rhinitis, conjunctivitis and cough/wheeze. Intensity of each symptom type was recorded individually (ranging from 0-10) and their sum (ranging from 0-50) is represented as the combined symptom scores. Data are presented as median [IQR].

### SuppInfo_Table 1. Detailed statistical data

### a) sIgG

### i. Der p 1

Table a.i.i: Levels sIgG: Der p 1 (µg/ml).

| **Time** | **Group** | **Mean** | **SD** | **Median** | **Q1** | **Q3** | **Min** | **Max** | **N** |
| --- | --- | --- | --- | --- | --- | --- | --- | --- | --- |
| **Baseline** | Placebo | 2.05 | 2.33 | 2.47 | 0 | 3.04 | 0 | 7.11 | 15 |
| **Baseline** | HDM-SLIT | 2.1 | 1.69 | 2.26 | 0.4 | 2.75 | 0 | 6.15 | 18 |
| **4 Months** | Placebo | 2.06 | 1.77 | 2.3 | 0 | 2.98 | 0 | 5.72 | 15 |
| **4 Months** | HDM-SLIT | 4.72 | 2.75 | 4.79 | 3.23 | 5.74 | 0 | 10.8 | 18 |
| **12 Months** | Placebo | 1.74 | 1.85 | 2.05 | 0 | 2.47 | 0 | 5.84 | 14 |
| **12 Months** | HDM-SLIT | 5.28 | 3.58 | 4.22 | 3.26 | 7.07 | 0 | 14.37 | 17 |
| **24 Months** | Placebo | 2.03 | 2.14 | 2.34 | 0 | 2.7 | 0 | 7.56 | 13 |
| **24 Months** | HDM-SLIT | 4.88 | 3.55 | 4.03 | 2.82 | 6.66 | 0 | 13.04 | 16 |

Table a.i.ii: Levels sIgG Der p 1: Linear Regression for change with time trend.

| **Subgroup** | **Comparison** | **Effect** | **Std Error** | **lower CL** | **upper CL** | **p-value** |
| --- | --- | --- | --- | --- | --- | --- |
| **Within Placebo** | 4 M vs. BL | 0.01 | 0.38 | -0.72 | 0.73 | 0.9 |
|  | 12 M vs. BL | -0.37 | 0.38 | -1.12 | 0.37 | 0.3 |
|  | 24 M vs. BL | -0.23 | 0.39 | -0.99 | 0.54 | 0.5 |
|  | 12 M vs. 4 M | -0.38 | 0.38 | -1.12 | 0.36 | 0.3 |
|  | 24 M vs. 4 M | -0.24 | 0.39 | -0.99 | 0.53 | 0.5 |
|  | 24 M vs. 12 M | 0.14 | 0.40 | -0.62 | 0.92 | 0.7 |
| **Within HDM-SLIT** | 4 M vs. BL | 2.63 | 0.72 | 1.23 | 4.03 | 0.001 |
|  | 12 M vs. BL | 3.21 | 0.73 | 1.77 | 4.62 | <0.001 |
|  | 24 M vs. BL | 2.89 | 0.75 | 1.41 | 4.32 | <0.001 |
|  | 12 M vs. 4 M | 0.59 | 0.73 | -0.85 | 2.00 | 0.4 |
|  | 24 M vs. 4 M | 0.26 | 0.75 | -1.21 | 1.69 | 0.7 |
|  | 24 M vs. 12 M | -0.33 | 0.76 | -1.81 | 1.14 | 0.6 |

Table a.i.iii: Levels sIgG Der p 1: Linear Regression for difference in change with time trend between groups.

| **Variable** | **Comparison** | **Effect** | **Std Error** | **lower CL** | **upper CL** | **p-value** |
| --- | --- | --- | --- | --- | --- | --- |
| **IA: Time*RD_Group** | 4 M vs. BL | 2.62 | 0.86 | 0.96 | 4.27 | 0.003 |
| **(HDM-SLIT vs. Placebo)** | 12 M vs. BL | 3.60 | 0.88 | 1.90 | 5.28 | <0.001 |
|  | 24 M vs. BL | 3.11 | 0.90 | 1.36 | 4.82 | 0.001 |

### ii. Der p-extract

Table a.ii.i: Levels sIgG: Der p-extract (µg/ml).

| **Time** | **Group** | **Mean** | **SD** | **Median** | **Q1** | **Q3** | **Min** | **Max** | **N** |
| --- | --- | --- | --- | --- | --- | --- | --- | --- | --- |
| **Baseline** | Placebo | 9.14 | 4.28 | 8.27 | 6.36 | 10.59 | 2.74 | 19.4 | 15 |
| **Baseline** | HDM-SLIT | 10.61 | 4.8 | 10.22 | 7.35 | 12.16 | 3.68 | 21.3 | 18 |
| **4 Months** | Placebo | 9.3 | 3.25 | 8.16 | 7.4 | 10.26 | 4.91 | 17.04 | 15 |
| **4 Months** | HDM-SLIT | 14.55 | 5.29 | 14.68 | 10.67 | 17.58 | 4.89 | 22.81 | 18 |
| **12 Months** | Placebo | 8.77 | 3.34 | 8.13 | 6.58 | 9.72 | 4.55 | 17.3 | 14 |
| **12 Months** | HDM-SLIT | 16.95 | 8.68 | 15.1 | 10.6 | 21.92 | 5.62 | 34.63 | 17 |
| **24 Months** | Placebo | 10.62 | 4.8 | 8.82 | 7.21 | 12.19 | 5.65 | 21.8 | 13 |
| **24 Months** | HDM-SLIT | 16.53 | 7.8 | 14.43 | 12.27 | 19.16 | 8.02 | 38.64 | 16 |

Table a.ii.ii: Levels sIgG Der p-extract: Linear Regression for change with time trend.

| **Subgroup** | **Comparison** | **Effect** | **Std Error** | **lower CL** | **upper CL** | **p-value** |
| --- | --- | --- | --- | --- | --- | --- |
| **Within Placebo** | 4 M vs. BL | 0.17 | 0.65 | -1.09 | 1.42 | 0.8 |
|  | 12 M vs. BL | -0.52 | 0.66 | -1.80 | 0.77 | 0.4 |
|  | 24 M vs. BL | 0.98 | 0.68 | -0.32 | 2.32 | 0.1 |
|  | 12 M vs. 4 M | -0.69 | 0.66 | -1.96 | 0.61 | 0.3 |
|  | 24 M vs. 4 M | 0.82 | 0.68 | -0.48 | 2.16 | 0.2 |
|  | 24 M vs. 12 M | 1.50 | 0.69 | 0.18 | 2.84 | 0.035 |
| **Within HDM-SLIT** | 4 M vs. BL | 3.94 | 1.47 | 1.08 | 6.79 | 0.01 |
|  | 12 M vs. BL | 6.46 | 1.50 | 3.54 | 9.35 | <0.001 |
|  | 24 M vs. BL | 6.63 | 1.53 | 3.62 | 9.55 | <0.001 |
|  | 12 M vs. 4 M | 2.52 | 1.50 | -0.40 | 5.42 | 0.09 |
|  | 24 M vs. 4 M | 2.69 | 1.53 | -0.32 | 5.61 | 0.085 |
|  | 24 M vs. 12 M | 0.17 | 1.56 | -2.88 | 3.16 | 0.9 |

Table a.ii.iii: Levels sIgG Der p-extract: Linear Regression for difference in change with time trend between groups.

| **Variable** | **Comparison** | **Effect** | **Std Error** | **lower CL** | **upper CL** | **p-value** |
| --- | --- | --- | --- | --- | --- | --- |
| **IA: Time*RD_Group** | 4 M vs. BL | 3.77 | 1.72 | 0.47 | 7.07 | 0.031 |
| **(HDM-SLIT vs. Placebo)** | 12 M vs. BL | 6.98 | 1.76 | 3.60 | 10.35 | <0.001 |
|  | 24 M vs. BL | 5.61 | 1.80 | 2.12 | 9.03 | 0.003 |

### iii. Der p 2

Table.a.iii.i: Levels sIgG: Der p 2 (µg/ml).

| **Time** | **Group** | **Mean** | **SD** | **Median** | **Q1** | **Q3** | **Min** | **Max** | **N** |
| --- | --- | --- | --- | --- | --- | --- | --- | --- | --- |
| **Baseline** | Placebo | 2.21 | 2.43 | 2.09 | 0 | 4.17 | 0 | 6.91 | 15 |
| **Baseline** | HDM-SLIT | 2.95 | 1.91 | 3.09 | 2.5 | 4.24 | 0 | 6.36 | 18 |
| **4 Months** | Placebo | 2.2 | 1.82 | 2.29 | 0 | 3.62 | 0 | 4.95 | 15 |
| **4 Months** | HDM-SLIT | 5.8 | 2.99 | 5.66 | 3.39 | 7.93 | 0 | 11.5 | 18 |
| **12 Months** | Placebo | 1.78 | 2.03 | 1.23 | 0 | 3.14 | 0 | 5.85 | 14 |
| **12 Months** | HDM-SLIT | 6.61 | 4.11 | 5.36 | 3.97 | 8.61 | 0 | 15.76 | 17 |
| **24 Months** | Placebo | 2.58 | 2.32 | 2.39 | 0 | 3.4 | 0 | 6.63 | 13 |
| **24 Months** | HDM-SLIT | 5.9 | 3.6 | 4.89 | 3.35 | 7.06 | 2.38 | 14.06 | 16 |

Table.a.iii.ii: Levels sIgG Der p 2: Linear Regression for change with time trend.

| **Subgroup** | **Comparison** | **Effect** | **Std Error** | **lower CL** | **upper CL** | **p-value** |
| --- | --- | --- | --- | --- | --- | --- |
| **Within Placebo** | 4 M vs. BL | -0.02 | 0.34 | -0.67 | 0.63 | 0.9 |
|  | 12 M vs. BL | -0.51 | 0.34 | -1.17 | 0.16 | 0.1 |
|  | 24 M vs. BL | 0.14 | 0.35 | -0.54 | 0.83 | 0.6 |
|  | 12 M vs. 4 M | -0.49 | 0.34 | -1.15 | 0.18 | 0.1 |
|  | 24 M vs. 4 M | 0.16 | 0.35 | -0.52 | 0.85 | 0.6 |
|  | 24 M vs. 12 M | 0.65 | 0.36 | -0.04 | 1.34 | 0.07 |
| **Within HDM-SLIT** | 4 M vs. BL | 2.85 | 0.70 | 1.48 | 4.21 | <0.001 |
|  | 12 M vs. BL | 3.80 | 0.72 | 2.40 | 5.19 | <0.001 |
|  | 24 M vs. BL | 3.16 | 0.73 | 1.73 | 4.57 | <0.001 |
|  | 12 M vs. 4 M | 0.95 | 0.72 | -0.45 | 2.34 | 0.1 |
|  | 24 M vs. 4 M | 0.31 | 0.73 | -1.12 | 1.72 | 0.6 |
|  | 24 M vs. 12 M | -0.64 | 0.75 | -2.09 | 0.80 | 0.3 |

Table.a.iii.iii: Levels sIgG Der p 2: Linear Regression for difference in change with time trend between groups.

| **Variable** | **Comparison** | **Effect** | **Std Error** | **lower CL** | **upper CL** | **p-value** |
| --- | --- | --- | --- | --- | --- | --- |
| **IA: Time*RD_Group** | 4 M vs. BL | 2.87 | 0.83 | 1.27 | 4.46 | 0.001 |
| **(HDM-SLIT vs. Placebo)** | 12 M vs. BL | 4.30 | 0.85 | 2.66 | 5.93 | <0.001 |
|  | 24 M vs. BL | 3.00 | 0.87 | 1.32 | 4.66 | 0.001 |

### iv. Der p 23

Table a.iv.i: Levels sIgG: Der p 23 (µg/ml).

| **Time** | **Group** | **Mean** | **SD** | **Median** | **Q1** | **Q3** | **Min** | **Max** | **N** |
| --- | --- | --- | --- | --- | --- | --- | --- | --- | --- |
| **Baseline** | Placebo | 0.73 | 1.34 | 0 | 0 | 1.04 | 0 | 4.2 | 15 |
| **Baseline** | HDM-SLIT | 0.53 | 1.03 | 0 | 0 | 0 | 0 | 2.55 | 18 |
| **4 Months** | Placebo | 0.38 | 1.03 | 0 | 0 | 0 | 0 | 3.52 | 15 |
| **4 Months** | HDM-SLIT | 2.05 | 2.08 | 2.53 | 0 | 3.38 | 0 | 5.8 | 18 |
| **12 Months** | Placebo | 0.31 | 1.17 | 0 | 0 | 0 | 0 | 4.36 | 14 |
| **12 Months** | HDM-SLIT | 1.61 | 1.94 | 0 | 0 | 3.16 | 0 | 5.34 | 17 |
| **24 Months** | Placebo | 0.32 | 1.16 | 0 | 0 | 0 | 0 | 4.18 | 13 |
| **24 Months** | HDM-SLIT | 1.3 | 1.4 | 1.01 | 0 | 2.5 | 0 | 3.58 | 16 |

Table a.iv.ii: Levels sIgG Der p 23: Median Regression for change with time trend.

| **Subgroup** | **Comparison** | **Effect** | **Std Error** | **lower CL** | **upper CL** | **p-value** |
| --- | --- | --- | --- | --- | --- | --- |
| **Within Placebo** | 4 M vs. BL | -0.37 | 0.23 | -0.84 | 0.10 | 0.1 |
|  | 12 M vs. BL | -0.42 | 0.27 | -0.97 | 0.12 | 0.1 |
|  | 24 M vs. BL | -0.41 | 0.27 | -0.96 | 0.13 | 0.1 |
|  | 12 M vs. 4 M | -0.06 | 0.15 | -0.37 | 0.24 | 0.6 |
|  | 24 M vs. 4 M | -0.05 | 0.15 | -0.36 | 0.25 | 0.7 |
|  | 24 M vs. 12 M | 0.02 | 0.06 | -0.09 | 0.13 | 0.7 |
| **Within HDM-SLIT** | 4 M vs. BL | 1.45 | 0.45 | 0.56 | 2.35 | 0.002 |
|  | 12 M vs. BL | 1.00 | 0.35 | 0.29 | 1.71 | 0.007 |
|  | 24 M vs. BL | 0.79 | 0.28 | 0.22 | 1.36 | 0.008 |
|  | 12 M vs. 4 M | -0.51 | 0.41 | -1.33 | 0.31 | 0.2 |
|  | 24 M vs. 4 M | -0.72 | 0.44 | -1.60 | 0.16 | 0.1 |
|  | 24 M vs. 12 M | -0.24 | 0.33 | -0.90 | 0.43 | 0.4 |

Table a.iv.iii: Levels sIgG Der p 23: Median Regression for difference in change with time trend between groups.

| **Variable** | **Comparison** | **Effect** | **Std Error** | **lower CL** | **upper CL** | **p-value** |
| --- | --- | --- | --- | --- | --- | --- |
| **IA: Time*RD_Group** | 4 M vs. BL | 1.85 | 0.55 | 0.76 | 2.95 | 0.001 |
| **(HDM-SLIT vs. Placebo)** | 12 M vs. BL | 1.46 | 0.55 | 0.35 | 2.57 | 0.011 |
|  | 24 M vs. BL | 1.22 | 0.34 | 0.54 | 1.89 | 0.001 |

### b) sIgG_1_

### i. Der p 1

Table b.i.i: Levels sIgG_1_: Der p 1 (µg/ml).

| **Time** | **Group** | **Mean** | **SD** | **Median** | **Q1** | **Q3** | **Min** | **Max** | **N** |
| --- | --- | --- | --- | --- | --- | --- | --- | --- | --- |
| **Baseline** | Placebo | 1.17 | 1.46 | 0.7 | 0.42 | 1.18 | 0.3 | 6.12 | 15 |
| **Baseline** | HDM-SLIT | 0.98 | 1.69 | 0.56 | 0.47 | 0.73 | 0.25 | 7.48 | 17 |
| **4 Months** | Placebo | 0.88 | 0.72 | 0.75 | 0.43 | 1.05 | 0.3 | 3.08 | 15 |
| **4 Months** | HDM-SLIT | 2.28 | 1.75 | 1.55 | 0.96 | 3.17 | 0.33 | 6.11 | 17 |
| **12 Months** | Placebo | 0.9 | 0.99 | 0.71 | 0.45 | 0.94 | 0.25 | 4.04 | 13 |
| **12 Months** | HDM-SLIT | 3.49 | 2.1 | 2.87 | 1.94 | 4.4 | 0.31 | 12 | 16 |
| **24 Months** | Placebo | 0.72 | 0.32 | 0.67 | 0.56 | 0.86 | 0.32 | 1.52 | 12 |
| **24 Months** | HDM-SLIT | 3.45 | 2.41 | 2.74 | 1.41 | 5.68 | 0.45 | 8.32 | 15 |

Table b.i.ii: Levels sIgG_1_ Der p 1: Linear Regression for change with time trend.

| **Subgroup** | **Comparison** | **Effect** | **Std Error** | **lower CL** | **upper CL** | **p-value** |
| --- | --- | --- | --- | --- | --- | --- |
| **Within Placebo** | 4 M vs. BL | -0.29 | 0.14 | -0.57 | -0.01 | 0.052 |
|  | 12 M vs. BL | -0.35 | 0.15 | -0.64 | -0.05 | 0.028 |
|  | 24 M vs. BL | -0.28 | 0.16 | -0.58 | 0.02 | 0.084 |
|  | 12 M vs. 4 M | -0.06 | 0.15 | -0.35 | 0.24 | 0.7 |
|  | 24 M vs. 4 M | 0.01 | 0.16 | -0.29 | 0.31 | 0.9 |
|  | 24 M vs. 12 M | 0.07 | 0.16 | -0.24 | 0.38 | 0.6 |
| **Within HDM-SLIT** | 4 M vs. BL | 1.30 | 0.60 | 0.14 | 2.45 | 0.035 |
|  | 12 M vs. BL | 2.52 | 0.61 | 1.32 | 3.68 | <0.001 |
|  | 24 M vs. BL | 2.48 | 0.62 | 1.27 | 3.67 | <0.001 |
|  | 12 M vs. 4 M | 1.22 | 0.61 | 0.03 | 2.39 | 0.05 |
|  | 24 M vs. 4 M | 1.18 | 0.62 | -0.03 | 2.37 | 0.063 |
|  | 24 M vs. 12 M | -0.04 | 0.63 | -1.26 | 1.19 | 0.9 |

Table b.i.iii: Levels sIgG_1_ Der p 1: Linear Regression for difference in change with time trend between groups.

| **Variable** | **Comparison** | **Effect** | **Std Error** | **lower CL** | **upper CL** | **p-value** |
| --- | --- | --- | --- | --- | --- | --- |
| **IA: Time*RD_Group** | 4 M vs. BL | 1.59 | 0.66 | 0.33 | 2.85 | 0.018 |
| **(HDM-SLIT vs. Placebo)** | 12 M vs. BL | 2.89 | 0.68 | 1.58 | 4.19 | <0.001 |
|  | 24 M vs. BL | 2.80 | 0.70 | 1.47 | 4.14 | <0.001 |

### ii. Der p-extract

Table b.ii.i: Levels sIgG_1_ Der p-extract (µg/ml).

| **Time** | **Group** | **Mean** | **SD** | **Median** | **Q1** | **Q3** | **Min** | **Max** | **N** |
| --- | --- | --- | --- | --- | --- | --- | --- | --- | --- |
| **Baseline** | Placebo | 0.77 | 0.78 | 0.43 | 0.34 | 0.82 | 0.17 | 2.81 | 15 |
| **Baseline** | HDM-SLIT | 0.97 | 1.19 | 0.71 | 0.44 | 0.82 | 0.2 | 5.26 | 17 |
| **4 Months** | Placebo | 0.9 | 0.68 | 0.75 | 0.4 | 1 | 0.22 | 2.22 | 15 |
| **4 Months** | HDM-SLIT | 2.56 | 1.24 | 2.5 | 1.9 | 3.57 | 0.46 | 4.57 | 17 |
| **12 Months** | Placebo | 0.95 | 0.76 | 0.71 | 0.65 | 1.06 | 0.17 | 3.13 | 13 |
| **12 Months** | HDM-SLIT | 3.34 | 2.39 | 2.92 | 2.1 | 3.68 | 0.9 | 11.3 | 16 |
| **24 Months** | Placebo | 0.82 | 0.55 | 0.69 | 0.56 | 0.94 | 0.21 | 2.29 | 12 |
| **24 Months** | HDM-SLIT | 3.06 | 1.78 | 2.92 | 1.62 | 3.7 | 1.06 | 6.94 | 15 |

Table b.ii.ii: Levels sIgG_1_ Der p-extract: Linear Regression for change with time trend.

| **Subgroup** | **Comparison** | **Effect** | **Std Error** | **lower CL** | **upper CL** | **p-value** |
| --- | --- | --- | --- | --- | --- | --- |
| **Within Placebo** | 4 M vs. BL | 0.13 | 0.13 | -0.12 | 0.39 | 0.3 |
|  | 12 M vs. BL | 0.12 | 0.14 | -0.15 | 0.39 | 0.4 |
|  | 24 M vs. BL | 0.15 | 0.14 | -0.13 | 0.43 | 0.2 |
|  | 12 M vs. 4 M | -0.02 | 0.14 | -0.28 | 0.26 | 0.9 |
|  | 24 M vs. 4 M | 0.02 | 0.14 | -0.26 | 0.29 | 0.9 |
|  | 24 M vs. 12 M | 0.03 | 0.15 | -0.26 | 0.31 | 0.8 |
| **Within HDM-SLIT** | 4 M vs. BL | 1.59 | 0.54 | 0.54 | 2.63 | 0.005 |
|  | 12 M vs. BL | 2.35 | 0.55 | 1.27 | 3.40 | <0.001 |
|  | 24 M vs. BL | 2.10 | 0.56 | 1.00 | 3.16 | <0.001 |
|  | 12 M vs. 4 M | 0.76 | 0.55 | -0.32 | 1.81 | 0.1 |
|  | 24 M vs. 4 M | 0.51 | 0.56 | -0.59 | 1.58 | 0.3 |
|  | 24 M vs. 12 M | -0.25 | 0.57 | -1.35 | 0.84 | 0.6 |

Table b.ii.iii: Levels sIgG_1_ Der p-extract: Linear Regression for difference in change with time trend between groups.

| **Variable** | **Comparison** | **Effect** | **Std Error** | **lower CL** | **upper CL** | **p-value** |
| --- | --- | --- | --- | --- | --- | --- |
| **IA: Time*RD_Group** | 4 M vs. BL | 1.45 | 0.60 | 0.31 | 2.59 | 0.017 |
| **(HDM-SLIT vs. Placebo)** | 12 M vs. BL | 2.23 | 0.62 | 1.05 | 3.40 | <0.001 |
|  | 24 M vs. BL | 2.00 | 0.63 | 0.80 | 3.21 | 0.002 |

### iii. Der p 2

Table b.iii.i: Levels sIgG_1_: Der p 2 (µg/ml).

| **Time** | **Group** | **Mean** | **SD** | **Median** | **Q1** | **Q3** | **Min** | **Max** | **N** |
| --- | --- | --- | --- | --- | --- | --- | --- | --- | --- |
| **Baseline** | Placebo | 0.89 | 0.9 | 0.63 | 0.37 | 0.93 | 0.17 | 3.04 | 15 |
| **Baseline** | HDM-SLIT | 1.02 | 1.06 | 0.78 | 0.52 | 1.04 | 0 | 4.55 | 17 |
| **4 Months** | Placebo | 0.84 | 0.65 | 0.6 | 0.44 | 0.96 | 0.08 | 2.22 | 15 |
| **4 Months** | HDM-SLIT | 2.59 | 1.64 | 2.73 | 1.45 | 3.04 | 0.12 | 5.53 | 17 |
| **12 Months** | Placebo | 0.8 | 0.69 | 0.67 | 0.3 | 0.97 | 0.08 | 2.55 | 13 |
| **12 Months** | HDM-SLIT | 3.02 | 2.23 | 2.68 | 1.61 | 3.32 | 0.94 | 9.68 | 16 |
| **24 Months** | Placebo | 0.64 | 0.31 | 0.67 | 0.47 | 0.77 | 0.08 | 1.25 | 12 |
| **24 Months** | HDM-SLIT | 2.42 | 1.81 | 1.88 | 1.34 | 2.78 | 0.62 | 7.28 | 15 |

Table b.iii.ii: Levels sIgG_1_ Der p 2: Linear Regression for change with time trend.

| **Subgroup** | **Comparison** | **Effect** | **Std Error** | **lower CL** | **upper CL** | **p-value** |
| --- | --- | --- | --- | --- | --- | --- |
| **Within Placebo** | 4 M vs. BL | -0.05 | 0.15 | -0.34 | 0.25 | 0.7 |
|  | 12 M vs. BL | -0.13 | 0.16 | -0.43 | 0.19 | 0.4 |
|  | 24 M vs. BL | -0.16 | 0.16 | -0.48 | 0.15 | 0.3 |
|  | 12 M vs. 4 M | -0.08 | 0.16 | -0.38 | 0.23 | 0.6 |
|  | 24 M vs. 4 M | -0.12 | 0.16 | -0.43 | 0.19 | 0.4 |
|  | 24 M vs. 12 M | -0.04 | 0.17 | -0.37 | 0.28 | 0.8 |
| **Within HDM-SLIT** | 4 M vs. BL | 1.57 | 0.51 | 0.59 | 2.55 | 0.003 |
|  | 12 M vs. BL | 1.99 | 0.52 | 0.99 | 2.98 | <0.001 |
|  | 24 M vs. BL | 1.44 | 0.53 | 0.41 | 2.44 | 0.009 |
|  | 12 M vs. 4 M | 0.42 | 0.52 | -0.58 | 1.41 | 0.42 |
|  | 24 M vs. 4 M | -0.13 | 0.53 | -1.16 | 0.87 | 0.8 |
|  | 24 M vs. 12 M | -0.55 | 0.53 | -1.59 | 0.47 | 0.3 |

Table b.iii.iii: Levels sIgG_1_ Der p 2: Linear Regression for difference in change with time trend between groups.

| **Variable** | **Comparison** | **Effect** | **Std Error** | **lower CL** | **upper CL** | **p-value** |
| --- | --- | --- | --- | --- | --- | --- |
| **IA: Time*RD_Group** | 4 M vs. BL | 1.61 | 0.56 | 0.54 | 2.69 | 0.005 |
| **(HDM-SLIT vs. Placebo)** | 12 M vs. BL | 2.11 | 0.58 | 0.99 | 3.22 | <0.001 |
|  | 24 M vs. BL | 1.63 | 0.60 | 0.48 | 2.76 | 0.008 |

### c) sIgG_4_

### i. Der p 1

Table c.i.i: Levels sIgG_4_: Der p 1 (µg/ml).

| **Time** | **Group** | **Mean** | **SD** | **Median** | **Q1** | **Q3** | **Min** | **Max** | **N** |
| --- | --- | --- | --- | --- | --- | --- | --- | --- | --- |
| **Baseline** | Placebo | 0.12 | 0.27 | 0 | 0 | 0 | 0 | 0.95 | 15 |
| **Baseline** | HDM-SLIT | 0.06 | 0.14 | 0 | 0 | 0 | 0 | 0.44 | 18 |
| **4 Months** | Placebo | 0.12 | 0.2 | 0 | 0 | 0.16 | 0 | 0.54 | 15 |
| **4 Months** | HDM-SLIT | 0.38 | 0.37 | 0.32 | 0 | 0.67 | 0 | 1.08 | 18 |
| **12 Months** | Placebo | 0.09 | 0.19 | 0 | 0 | 0 | 0 | 0.57 | 14 |
| **12 Months** | HDM-SLIT | 0.44 | 0.49 | 0.33 | 0 | 0.77 | 0 | 1.46 | 17 |
| **24 Months** | Placebo | 0.12 | 0.26 | 0 | 0 | 0 | 0 | 0.87 | 13 |
| **24 Months** | HDM-SLIT | 0.22 | 0.33 | 0 | 0 | 0.45 | 0 | 1.05 | 16 |

Table c.i.ii: Levels sIgG_4_ Der p 1: Median Regression for change with time trend.

| **Subgroup** | **Comparison** | **Effect** | **Std Error** | **lower CL** | **upper CL** | **p-value** |
| --- | --- | --- | --- | --- | --- | --- |
| **Within Placebo** | 4 M vs. BL | 0.00 | 0.04 | -0.08 | 0.08 | 0.9 |
|  | 12 M vs. BL | -0.02 | 0.04 | -0.10 | 0.05 | 0.5 |
|  | 24 M vs. BL | 0.01 | 0.03 | -0.06 | 0.08 | 0.8 |
|  | 12 M vs. 4 M | -0.02 | 0.03 | -0.08 | 0.04 | 0.4 |
|  | 24 M vs. 4 M | 0.01 | 0.05 | -0.09 | 0.10 | 0.8 |
|  | 24 M vs. 12 M | 0.03 | 0.05 | -0.06 | 0.13 | 0.5 |
| **Within HDM-SLIT** | 4 M vs. BL | 0.31 | 0.08 | 0.16 | 0.47 | <0.001 |
|  | 12 M vs. BL | 0.38 | 0.12 | 0.14 | 0.62 | 0.002 |
|  | 24 M vs. BL | 0.15 | 0.07 | 0.01 | 0.29 | 0.031 |
|  | 12 M vs. 4 M | 0.06 | 0.08 | -0.10 | 0.23 | 0.4 |
|  | 24 M vs. 4 M | -0.17 | 0.06 | -0.30 | -0.03 | 0.014 |
|  | 24 M vs. 12 M | -0.23 | 0.11 | -0.44 | -0.02 | 0.033 |

Table c.i.iii: Levels sIgG_4_ Der p 1: Median Regression for difference in change with time trend between groups.

| **Variable** | **Comparison** | **Effect** | **Std Error** | **lower CL** | **upper CL** | **p-value** |
| --- | --- | --- | --- | --- | --- | --- |
| **IA: Time*RD_Group** | 4 M vs. BL | 0.31 | 0.08 | 0.15 | 0.48 | <0.001 |
| **(HDM-SLIT vs. Placebo)** | 12 M vs. BL | 0.40 | 0.10 | 0.20 | 0.60 | <0.001 |
|  | 24 M vs. BL | 0.14 | 0.07 | 0.01 | 0.28 | 0.041 |

### ii. Der p-extract

Table c.ii.i: Levels sIgG_4_: Der p-extract (µg/ml).

| **Time** | **Group** | **Mean** | **SD** | **Median** | **Q1** | **Q3** | **Min** | **Max** | **N** |
| --- | --- | --- | --- | --- | --- | --- | --- | --- | --- |
| **Baseline** | Placebo | 0.28 | 0.62 | 0 | 0 | 0.15 | 0 | 2.14 | 15 |
| **Baseline** | HDM-SLIT | 0.22 | 0.29 | 0 | 0 | 0.37 | 0 | 0.86 | 18 |
| **4 Months** | Placebo | 0.26 | 0.43 | 0 | 0 | 0.52 | 0 | 1.3 | 15 |
| **4 Months** | HDM-SLIT | 1.02 | 0.84 | 0.9 | 0.44 | 1.45 | 0 | 3.39 | 18 |
| **12 Months** | Placebo | 0.33 | 0.54 | 0 | 0 | 0.51 | 0 | 1.95 | 14 |
| **12 Months** | HDM-SLIT | 1.41 | 1.23 | 0.91 | 0.77 | 1.93 | 0 | 4.83 | 17 |
| **24 Months** | Placebo | 0.4 | 0.71 | 0 | 0 | 0.59 | 0 | 2.41 | 13 |
| **24 Months** | HDM-SLIT | 1.17 | 0.83 | 1.11 | 0.62 | 1.63 | 0 | 3.07 | 16 |

Table c.ii.ii: Levels sIgG_4_ Der p-extract: Median Regression for change with time trend.

| **Subgroup** | **Comparison** | **Effect** | **Std Error** | **lower CL** | **upper CL** | **p-value** |
| --- | --- | --- | --- | --- | --- | --- |
| **Within Placebo** | 4 M vs. BL | 0.00 | 0.07 | -0.13 | 0.14 | 0.9 |
|  | 12 M vs. BL | 0.02 | 0.07 | -0.12 | 0.16 | 0.7 |
|  | 24 M vs. BL | 0.10 | 0.09 | -0.07 | 0.27 | 0.2 |
|  | 12 M vs. 4 M | 0.04 | 0.08 | -0.12 | 0.19 | 0.6 |
|  | 24 M vs. 4 M | 0.11 | 0.12 | -0.13 | 0.35 | 0.3 |
|  | 24 M vs. 12 M | 0.04 | 0.08 | -0.13 | 0.20 | 0.6 |
| **Within HDM-SLIT** | 4 M vs. BL | 0.80 | 0.16 | 0.47 | 1.13 | <0.001 |
|  | 12 M vs. BL | 1.16 | 0.35 | 0.46 | 1.86 | 0.002 |
|  | 24 M vs. BL | 0.97 | 0.19 | 0.59 | 1.35 | <0.001 |
|  | 12 M vs. 4 M | 0.37 | 0.24 | -0.13 | 0.86 | 0.1 |
|  | 24 M vs. 4 M | 0.17 | 0.19 | -0.21 | 0.56 | 0.3 |
|  | 24 M vs. 12 M | -0.24 | 0.34 | -0.93 | 0.46 | 0.4 |

Table c.ii.iii: Levels sIgG_4_ Der p-extract: Median Regression for difference in change with time trend between groups.

| **Variable** | **Comparison** | **Effect** | **Std Error** | **lower CL** | **upper CL** | **p-value** |
| --- | --- | --- | --- | --- | --- | --- |
| **IA: Time*RD_Group** | 4 M vs. BL | 0.79 | 0.16 | 0.48 | 1.11 | <0.001 |
| **(HDM-SLIT vs. Placebo)** | 12 M vs. BL | 1.13 | 0.31 | 0.51 | 1.75 | 0.001 |
|  | 24 M vs. BL | 0.80 | 0.22 | 0.36 | 1.24 | 0.001 |

### iii. Der p 2

Table c.iii.i: Levels sIgG_4_: Der p 2 (µg/ml).

| **Time** | **Group** | **Mean** | **SD** | **Median** | **Q1** | **Q3** | **Min** | **Max** | **N** |
| --- | --- | --- | --- | --- | --- | --- | --- | --- | --- |
| **Baseline** | Placebo | 0.17 | 0.42 | 0 | 0 | 0 | 0 | 1.47 | 15 |
| **Baseline** | HDM-SLIT | 0.06 | 0.18 | 0 | 0 | 0 | 0 | 0.69 | 18 |
| **4 Months** | Placebo | 0.12 | 0.27 | 0 | 0 | 0 | 0 | 0.87 | 15 |
| **4 Months** | HDM-SLIT | 0.56 | 0.69 | 0.39 | 0 | 0.75 | 0 | 2.46 | 18 |
| **12 Months** | Placebo | 0.12 | 0.25 | 0 | 0 | 0 | 0 | 0.75 | 14 |
| **12 Months** | HDM-SLIT | 0.71 | 0.75 | 0.61 | 0 | 0.9 | 0 | 2.57 | 17 |
| **24 Months** | Placebo | 0.2 | 0.34 | 0 | 0 | 0.45 | 0 | 0.98 | 13 |
| **24 Months** | HDM-SLIT | 0.51 | 0.48 | 0.43 | 0 | 0.79 | 0 | 1.6 | 16 |

Table c.iii.ii: Levels sIgG_4_ Der p 2: Median Regression for change with time trend.

| **Subgroup** | **Comparison** | **Effect** | **Std Error** | **lower CL** | **upper CL** | **p-value** |
| --- | --- | --- | --- | --- | --- | --- |
| **Within Placebo** | 4 M vs. BL | -0.06 | 0.04 | -0.13 | 0.02 | 0.1 |
|  | 12 M vs. BL | -0.06 | 0.06 | -0.19 | 0.07 | 0.3 |
|  | 24 M vs. BL | 0.02 | 0.08 | -0.13 | 0.18 | 0.7 |
|  | 12 M vs. 4 M | -0.01 | 0.04 | -0.10 | 0.08 | 0.8 |
|  | 24 M vs. 4 M | 0.07 | 0.07 | -0.07 | 0.22 | 0.3 |
|  | 24 M vs. 12 M | 0.08 | 0.06 | -0.04 | 0.20 | 0.1 |
| **Within HDM-SLIT** | 4 M vs. BL | 0.40 | 0.17 | 0.07 | 0.74 | 0.018 |
|  | 12 M vs. BL | 0.63 | 0.23 | 0.17 | 1.09 | 0.009 |
|  | 24 M vs. BL | 0.54 | 0.16 | 0.21 | 0.87 | 0.002 |
|  | 12 M vs. 4 M | 0.14 | 0.13 | -0.13 | 0.40 | 0.2 |
|  | 24 M vs. 4 M | 0.02 | 0.14 | -0.25 | 0.30 | 0.8 |
|  | 24 M vs. 12 M | -0.14 | 0.15 | -0.45 | 0.17 | 0.3 |

Table c.iii.iii: Levels sIgG_4_ Der p 2: Median Regression for difference in change with time trend between groups.

| **Variable** | **Comparison** | **Effect** | **Std Error** | **lower CL** | **upper CL** | **p-value** |
| --- | --- | --- | --- | --- | --- | --- |
| **IA: Time*RD_Group** | 4 M vs. BL | 0.51 | 0.14 | 0.23 | 0.80 | 0.001 |
| **(HDM-SLIT vs. Placebo)** | 12 M vs. BL | 0.70 | 0.19 | 0.32 | 1.09 | 0.001 |
|  | 24 M vs. BL | 0.41 | 0.14 | 0.13 | 0.69 | 0.005 |

### iv. Der p 23

Table c.iv.i: Levels sIgG_4_: Der p 23 (µg/ml).

| **Time** | **Group** | **Mean** | **SD** | **Median** | **Q1** | **Q3** | **Min** | **Max** | **N** |
| --- | --- | --- | --- | --- | --- | --- | --- | --- | --- |
| **Baseline** | Placebo | 0.05 | 0.21 | 0 | 0 | 0 | 0 | 0.81 | 15 |
| **Baseline** | HDM-SLIT | 0 | 0 | 0 | 0 | 0 | 0 | 0 | 18 |
| **4 Months** | Placebo | 0.04 | 0.14 | 0 | 0 | 0 | 0 | 0.56 | 15 |
| **4 Months** | HDM-SLIT | 0.15 | 0.28 | 0 | 0 | 0 | 0 | 0.71 | 18 |
| **12 Months** | Placebo | 0.06 | 0.22 | 0 | 0 | 0 | 0 | 0.84 | 14 |
| **12 Months** | HDM-SLIT | 0.15 | 0.38 | 0 | 0 | 0 | 0 | 1.37 | 17 |
| **24 Months** | Placebo | 0.04 | 0.15 | 0 | 0 | 0 | 0 | 0.54 | 13 |
| **24 Months** | HDM-SLIT | 0.05 | 0.13 | 0 | 0 | 0 | 0 | 0.44 | 16 |

Table c.iv.ii: Levels sIgG_4_ Der p 23: Median Regression for change with time trend.

| **Subgroup** | **Comparison** | **Effect** | **Std Error** | **lower CL** | **upper CL** | **p-value** |
| --- | --- | --- | --- | --- | --- | --- |
| **Within Placebo** | 4 M vs. BL | -0.01 | 0.01 | -0.04 | 0.02 | 0.4 |
|  | 12 M vs. BL | 0.00 | 0.01 | -0.02 | 0.02 | 0.9 |
|  | 24 M vs. BL | -0.01 | 0.01 | -0.04 | 0.02 | 0.5 |
|  | 12 M vs. 4 M | 0.02 | 0.02 | -0.02 | 0.06 | 0.3 |
|  | 24 M vs. 4 M | 0.01 | 0.01 | -0.01 | 0.02 | 0.4 |
|  | 24 M vs. 12 M | -0.02 | 0.02 | -0.06 | 0.02 | 0.3 |
| **Within HDM-SLIT** | 4 M vs. BL | 0.07 | 0.07 | -0.07 | 0.22 | 0.3 |
|  | 12 M vs. BL | 0.07 | 0.10 | -0.12 | 0.27 | 0.4 |
|  | 24 M vs. BL | 0.06 | 0.04 | -0.02 | 0.14 | 0.1 |
|  | 12 M vs. 4 M | -0.03 | 0.05 | -0.13 | 0.07 | 0.5 |
|  | 24 M vs. 4 M | -0.07 | 0.05 | -0.17 | 0.02 | 0.1 |
|  | 24 M vs. 12 M | -0.09 | 0.07 | -0.23 | 0.06 | 0.2 |

Table c.iv.iii: Levels sIgG_4_ Der p 23: Median Regression for difference in change with time trend between groups.

| **Variable** | **Comparison** | **Effect** | **Std Error** | **lower CL** | **upper CL** | **p-value** |
| --- | --- | --- | --- | --- | --- | --- |
| **IA: Time*RD_Group** | 4 M vs. BL | 0.14 | 0.08 | -0.03 | 0.30 | 0.097 |
| **(HDM-SLIT vs. Placebo)** | 12 M vs. BL | 0.13 | 0.11 | -0.09 | 0.35 | 0.2 |
|  | 24 M vs. BL | 0.05 | 0.04 | -0.02 | 0.13 | 0.1 |

## d) **Levels of sIgG to non-treatment allergens**

## i. ***Ambrosia***

Table d.i.i: Levels IgG: *Ambrosia* (µg/ml).

| **Time** | **Group** | **Mean** | **SD** | **Median** | **Q1** | **Q3** | **Min** | **Max** | **N** |
| --- | --- | --- | --- | --- | --- | --- | --- | --- | --- |
| **Baseline** | Placebo | 1.38 | 1.5 | 1.81 | 0 | 2.28 | 0 | 4.86 | 15 |
| **Baseline** | HDM-SLIT | 1.8 | 1.93 | 1.19 | 0 | 3.5 | 0 | 4.66 | 18 |
| **4 Months** | Placebo | 0.88 | 1.33 | 0 | 0 | 2.16 | 0 | 3.74 | 15 |
| **4 Months** | HDM-SLIT | 1.95 | 1.87 | 2.72 | 0 | 3.51 | 0 | 5.2 | 18 |
| **12 Months** | Placebo | 1.16 | 1.44 | 0 | 0 | 2.49 | 0 | 3.71 | 14 |
| **12 Months** | HDM-SLIT | 2.12 | 1.87 | 2.3 | 0 | 3.29 | 0 | 5.91 | 17 |
| **24 Months** | Placebo | 1.54 | 1.77 | 0 | 0 | 3.03 | 0 | 4.03 | 13 |
| **24 Months** | HDM-SLIT | 1.87 | 1.66 | 2.24 | 0 | 2.75 | 0 | 4.69 | 16 |

Table d.i.ii: Levels IgG *Ambrosia*: Median Regression for change with time trend.

| **Subgroup** | **Comparison** | **Effekt** | **Std Error** | **lower CL** | **upper CL** | **p-value** |
| --- | --- | --- | --- | --- | --- | --- |
| **Within Placebo** | 4 M vs. BL | -0.40 | 0.22 | -0.85 | 0.05 | 0.08 |
|  | 12 M vs. BL | -0.08 | 0.23 | -0.54 | 0.38 | 0.7 |
|  | 24 M vs. BL | 0.06 | 0.28 | -0.49 | 0.62 | 0.8 |
|  | 12 M vs. 4 M | 0.36 | 0.14 | 0.08 | 0.65 | 0.01 |
|  | 24 M vs. 4 M | 0.58 | 0.25 | 0.07 | 1.09 | 0.03 |
|  | 24 M vs. 12 M | 0.28 | 0.20 | -0.13 | 0.69 | 0.2 |
| **Within HDM-SLIT** | 4 M vs. BL | 0.11 | 0.22 | -0.33 | 0.55 | 0.6 |
|  | 12 M vs. BL | 0.25 | 0.28 | -0.32 | 0.81 | 0.4 |
|  | 24 M vs. BL | 0.11 | 0.24 | -0.37 | 0.59 | 0.65 |
|  | 12 M vs. 4 M | 0.08 | 0.19 | -0.31 | 0.47 | 0.7 |
|  | 24 M vs. 4 M | -0.02 | 0.17 | -0.36 | 0.33 | 0.9 |
|  | 24 M vs. 12 M | -0.17 | 0.25 | -0.67 | 0.33 | 0.5 |

Table d.i.iii: Levels IgG *Ambrosia*: Median Regression for difference in change with time trend between groups.

| **Variable** | **Comparison** | **Effekt** | **Std Error** | **lower CL** | **upper CL** | **p-value** |
| --- | --- | --- | --- | --- | --- | --- |
| **IA: Time*RD_Group** | 4 M vs. BL | 0.64 | 0.30 | 0.03 | 1.25 | 0.04 |
| **(HDM-SLIT vs. Placebo)** | 12 M vs. BL | 0.50 | 0.39 | -0.29 | 1.29 | 0.2 |
|  | 24 M vs. BL | -0.01 | 0.45 | -0.91 | 0.88 | 1.0 |

## ii. **Dog dander**

Table d.ii.i: Levels IgG: dog dander (µg/ml).

| **Time** | **Group** | **Mean** | **SD** | **Median** | **Q1** | **Q3** | **Min** | **Max** | **N** |
| --- | --- | --- | --- | --- | --- | --- | --- | --- | --- |
| **Baseline** | Placebo | 5.98 | 2.89 | 5.85 | 4.24 | 8.14 | 0 | 9.79 | 15 |
| **Baseline** | HDM-SLIT | 8.1 | 3.77 | 7.31 | 5.67 | 9.39 | 3.38 | 17.63 | 18 |
| **4 Months** | Placebo | 6.02 | 2.32 | 5.47 | 4.62 | 7.67 | 2.97 | 10.4 | 15 |
| **4 Months** | HDM-SLIT | 7.97 | 3.63 | 7.86 | 5.28 | 8.69 | 2.27 | 17.39 | 18 |
| **12 Months** | Placebo | 5.89 | 2.6 | 5.03 | 4.05 | 7.94 | 2.54 | 10.1 | 14 |
| **12 Months** | HDM-SLIT | 7.82 | 4.57 | 6.03 | 4.89 | 9.15 | 3.31 | 18.25 | 17 |
| **24 Months** | Placebo | 7.35 | 3.53 | 6.99 | 5.15 | 8.67 | 3.07 | 15.36 | 13 |
| **24 Months** | HDM-SLIT | 7.31 | 3.2 | 6.82 | 5.3 | 8.67 | 2.95 | 14.13 | 16 |

Table d.ii.ii: Levels IgG dog dander: Linear Regression for change with time trend.

| **Subgroup** | **Comparison** | **Effekt** | **Std Error** | **lower CL** | **upper CL** | **p-value** |
| --- | --- | --- | --- | --- | --- | --- |
| **Within Placebo** | 4 M vs. BL | 0.03 | 0.51 | -0.96 | 1.03 | 0.9 |
|  | 12 M vs. BL | -0.12 | 0.53 | -1.14 | 0.90 | 0.8 |
|  | 24 M vs. BL | 1.10 | 0.54 | 0.07 | 2.15 | 0.05 |
|  | 12 M vs. 4 M | -0.15 | 0.53 | -1.17 | 0.87 | 0.7 |
|  | 24 M vs. 4 M | 1.07 | 0.54 | 0.03 | 2.12 | 0.05 |
|  | 24 M vs. 12 M | 1.22 | 0.55 | 0.18 | 2.28 | 0.03 |
| **Within HDM-SLIT** | 4 M vs. BL | -0.13 | 0.35 | -0.82 | 0.55 | 0.7 |
|  | 12 M vs. BL | -0.24 | 0.36 | -0.94 | 0.46 | 0.5 |
|  | 24 M vs. BL | -0.11 | 0.37 | -0.83 | 0.60 | 0.8 |
|  | 12 M vs. 4 M | -0.11 | 0.36 | -0.81 | 0.59 | 0.8 |
|  | 24 M vs. 4 M | 0.03 | 0.37 | -0.69 | 0.74 | 0.9 |
|  | 24 M vs. 12 M | 0.13 | 0.38 | -0.60 | 0.86 | 0.7 |

Table d.ii.iii: Levels IgG dog dander: Linear Regression for difference in change with time trend between groups.

| **Variable** | **Comparison** | **Effekt** | **Std Error** | **lower CL** | **upper CL** | **p-value** |
| --- | --- | --- | --- | --- | --- | --- |
| **IA: Time*RD_Group** | 4 M vs. BL | -0.17 | 0.61 | -1.33 | 1.00 | 0.7 |
| **(HDM-SLIT vs. Placebo)** | 12 M vs. BL | -0.13 | 0.62 | -1.32 | 1.07 | 0.8 |
|  | 24 M vs. BL | -1.21 | 0.64 | -2.44 | 0.00 | 0.06 |

## iii. **Walnut**

Table d.iii.i: Levels sIgG: Walnut (µg/ml).

| **Time** | **Group** | **Mean** | **SD** | **Median** | **Q1** | **Q3** | **Min** | **Max** | **N** |
| --- | --- | --- | --- | --- | --- | --- | --- | --- | --- |
| **Baseline** | Placebo | 1.18 | 1.35 | 0 | 0 | 2.42 | 0 | 3.58 | 15 |
| **Baseline** | HDM-SLIT | 2.58 | 2.65 | 2.82 | 0 | 3.61 | 0 | 9.4 | 18 |
| **4 Months** | Placebo | 0.68 | 1.18 | 0 | 0 | 1.09 | 0 | 2.86 | 15 |
| **4 Months** | HDM-SLIT | 2.75 | 2.81 | 2.53 | 0 | 4.19 | 0 | 9.55 | 18 |
| **12 Months** | Placebo | 0.72 | 1.2 | 0 | 0 | 1.61 | 0 | 2.95 | 14 |
| **12 Months** | HDM-SLIT | 2.84 | 3.3 | 2.68 | 0 | 3.84 | 0 | 10.93 | 17 |
| **24 Months** | Placebo | 1.52 | 1.77 | 0 | 0 | 3.4 | 0 | 4.07 | 13 |
| **24 Months** | HDM-SLIT | 2.22 | 2.34 | 2.27 | 0 | 3.3 | 0 | 6.54 | 16 |

Table d.iii.ii: Levels sIgG Walnut: Median Regression for change with time trend.

| **Subgroup** | **Comparison** | **Effect** | **Std Error** | **lower CL** | **upper CL** | **p-value** |
| --- | --- | --- | --- | --- | --- | --- |
| **Within Placebo** | 4 M vs. BL | 0.02 | 0.01 | 0.00 | 0.03 | 0.009 |
|  | 12 M vs. BL | 0.02 | 0.04 | -0.07 | 0.11 | 0.6 |
|  | 24 M vs. BL | 0.10 | 0.09 | -0.08 | 0.27 | 0.2 |
|  | 12 M vs. 4 M | 0.02 | 0.04 | -0.07 | 0.11 | 0.6 |
|  | 24 M vs. 4 M | 0.10 | 0.09 | -0.08 | 0.27 | 0.2 |
|  | 24 M vs. 12 M | 0.05 | 0.05 | -0.05 | 0.16 | 0.3 |
| **Within HDM-SLIT** | 4 M vs. BL | 0.05 | 0.08 | -0.10 | 0.21 | 0.4 |
|  | 12 M vs. BL | 0.11 | 0.12 | -0.12 | 0.34 | 0.3 |
|  | 24 M vs. BL | 0.01 | 0.11 | -0.22 | 0.23 | 0.9 |
|  | 12 M vs. 4 M | 0.01 | 0.05 | -0.08 | 0.11 | 0.7 |
|  | 24 M vs. 4 M | -0.13 | 0.17 | -0.46 | 0.20 | 0.4 |
|  | 24 M vs. 12 M | -0.19 | 0.22 | -0.63 | 0.25 | 0.3 |

Table d.iii.iii: Levels sIgG Walnut: Median Regression for difference in change with time trend between groups.

| **Variable** | **Comparison** | **Effect** | **Std Error** | **lower CL** | **upper CL** | **p-value** |
| --- | --- | --- | --- | --- | --- | --- |
| **IA: Time*RD_Group** | 4 M vs. BL | 0.04 | 0.07 | -0.11 | 0.19 | 0.6 |
| **(HDM-SLIT vs. Placebo)** | 12 M vs. BL | 0.04 | 0.12 | -0.19 | 0.27 | 0.7 |
|  | 24 M vs. BL | -0.22 | 0.17 | -0.55 | 0.12 | 0.2 |

## iv. ***Aspergillus***

Table d.iv.i: Levels sIgG: *Aspergillus* (µg/ml)*.*

| **Time** | **Group** | **Mean** | **SD** | **Median** | **Q1** | **Q3** | **Min** | **Max** | **N** |
| --- | --- | --- | --- | --- | --- | --- | --- | --- | --- |
| **Baseline** | Placebo | 7.32 | 4.17 | 6.98 | 4.56 | 8.86 | 2.39 | 18.41 | 15 |
| **Baseline** | HDM-SLIT | 8.98 | 6.5 | 6.97 | 5.59 | 9.96 | 0 | 26.1 | 18 |
| **4 Months** | Placebo | 8.61 | 5.35 | 6.56 | 5.22 | 10.09 | 3.4 | 23.45 | 15 |
| **4 Months** | HDM-SLIT | 9.88 | 6.28 | 8.86 | 5.31 | 14.28 | 0 | 23.18 | 18 |
| **12 Months** | Placebo | 8.39 | 4.77 | 7.11 | 5.54 | 9.48 | 3.17 | 22.24 | 14 |
| **12 Months** | HDM-SLIT | 10.21 | 8.15 | 6.93 | 5.48 | 14.7 | 0 | 30.57 | 17 |
| **24 Months** | Placebo | 15.41 | 11.79 | 12.6 | 5.55 | 16.4 | 4.45 | 39.43 | 13 |
| **24 Months** | HDM-SLIT | 14.92 | 12.59 | 10.24 | 7.25 | 15.91 | 3.27 | 44.07 | 16 |

Table d.iv.ii: Levels sIgG *Aspergillus*: Linear Regression for change with time trend.

| **Subgroup** | **Comparison** | **Effect** | **Std Error** | **lower CL** | **upper CL** | **p-value** |
| --- | --- | --- | --- | --- | --- | --- |
| **Within Placebo** | 4 M vs. BL | 1.29 | 1.62 | -1.84 | 4.43 | 0.4 |
|  | 12 M vs. BL | 1.03 | 1.66 | -2.13 | 4.29 | 0.5 |
|  | 24 M vs. BL | 7.74 | 1.70 | 4.49 | 11.06 | <0.001 |
|  | 12 M vs. 4 M | -0.26 | 1.66 | -3.42 | 2.99 | 0.8 |
|  | 24 M vs. 4 M | 6.44 | 1.70 | 3.20 | 9.77 | 0.001 |
|  | 24 M vs. 12 M | 6.70 | 1.72 | 3.38 | 10.02 | <0.001 |
| **Within HDM-SLIT** | 4 M vs. BL | 0.90 | 1.61 | -2.22 | 4.02 | 0.5 |
|  | 12 M vs. BL | 1.48 | 1.64 | -1.70 | 4.65 | 0.3 |
|  | 24 M vs. BL | 6.58 | 1.67 | 3.32 | 9.80 | <0.001 |
|  | 12 M vs. 4 M | 0.58 | 1.64 | -2.60 | 3.75 | 0.7 |
|  | 24 M vs. 4 M | 5.68 | 1.67 | 2.42 | 8.90 | 0.001 |
|  | 24 M vs. 12 M | 5.10 | 1.70 | 1.78 | 8.38 | 0.004 |

Table d.iv.iii: Levels sIgG *Aspergillus*: Linear Regression for difference in change with time trend between groups.

| **Variable** | **Comparison** | **Effect** | **Std Error** | **lower CL** | **upper CL** | **p-value** |
| --- | --- | --- | --- | --- | --- | --- |
| **IA: Time*RD_Group** | 4 M vs. BL | -0.39 | 2.30 | -4.80 | 4.01 | 0.8 |
| **(HDM-SLIT vs. Placebo)** | 12 M vs. BL | 0.52 | 2.35 | -3.99 | 5.01 | 0.8 |
|  | 24 M vs. BL | -1.12 | 2.40 | -5.76 | 3.46 | 0.6 |

## e) **sIgE levels**

## i. **Der p-extract**

Table e.i.i: sIgE: Der p-extract (µg/ml).

| **Time** | **Group** | **Mean** | **SD** | **Median** | **Q1** | **Q3** | **Min** | **Max** | **N** |
| --- | --- | --- | --- | --- | --- | --- | --- | --- | --- |
| **Baseline** | Placebo | 26.08 | 19.03 | 21.7 | 13.9 | 40.2 | 0.64 | 58.7 | 15 |
| **Baseline** | HDM-SLIT | 61.61 | 141.99 | 21.3 | 9.15 | 46.78 | 1.42 | 618 | 18 |
| **4 Months** | Placebo | 48.63 | 72 | 27.9 | 15.45 | 49.2 | 0.73 | 292 | 15 |
| **4 Months** | HDM-SLIT | 136.57 | 248.21 | 43.85 | 23.1 | 83.07 | 6 | 976 | 18 |
| **12 Months** | Placebo | 52.43 | 80.34 | 31.65 | 15.4 | 51.2 | 0.65 | 319 | 14 |
| **12 Months** | HDM-SLIT | 105.83 | 199.21 | 38.05 | 14.75 | 108.72 | 1.13 | 861 | 18 |
| **24 Months** | Placebo | 92.48 | 186.02 | 47.8 | 15.6 | 72.5 | 0.46 | 703 | 13 |
| **24 Months** | HDM-SLIT | 45.13 | 81.31 | 19.45 | 14.1 | 38.55 | 0.68 | 339 | 16 |

Table e.i.ii: sIgE Der p-extract: Median Regression for change with time trend.

| **Subgroup** | **Comparison** | **Effect** | **Std Error** | **lower CL** | **upper CL** | **p-value** |
| --- | --- | --- | --- | --- | --- | --- |
| **Within Placebo** | 4 M vs. BL | 15.13 | 14.88 | -14.77 | 45.03 | 0.3 |
|  | 12 M vs. BL | 15.53 | 17.77 | -20.17 | 51.24 | 0.3 |
|  | 24 M vs. BL | 29.54 | 33.50 | -37.79 | 96.87 | 0.3 |
|  | 12 M vs. 4 M | 1.55 | 6.99 | -12.50 | 15.60 | 0.8 |
|  | 24 M vs. 4 M | 17.83 | 21.01 | -24.40 | 60.06 | 0.4 |
|  | 24 M vs. 12 M | 12.38 | 18.33 | -24.45 | 49.21 | 0.5 |
| **Within HDM-SLIT** | 4 M vs. BL | 13.99 | 37.90 | -62.18 | 90.16 | 0.7 |
|  | 12 M vs. BL | 9.29 | 41.21 | -73.53 | 92.11 | 0.8 |
|  | 24 M vs. BL | -1.99 | 38.00 | -78.35 | 74.37 | 0.9 |
|  | 12 M vs. 4 M | -27.96 | 23.67 | -75.54 | 19.62 | 0.2 |
|  | 24 M vs. 4 M | -47.86 | 34.29 | -116.77 | 21.04 | 0.1 |
|  | 24 M vs. 12 M | -32.85 | 24.12 | -81.34 | 5.63 | 0.1 |

Table e.i.iii: sIgE Der p-extract: Median Regression for difference in change with time trend between groups.

| **Variable** | **Comparison** | **Effect** | **Std Error** | **lower CL** | **upper CL** | **p-value** |
| --- | --- | --- | --- | --- | --- | --- |
| **IA: Time*RD_Group** | 4 M vs. BL | 17.98 | 40.22 | -62.85 | 98.80 | 0.6 |
| **(HDM-SLIT vs. Placebo)** | 12 M vs. BL | 1.09 | 40.26 | -79.82 | 81.99 | 0.9 |
|  | 24 M vs. BL | -64.30 | 51.19 | -167.17 | 38.58 | 0.2 |

## ii. **Der f-extract**

Table e.ii.i: sIgE: Der f-extract (µg/ml).

| **Time** | **Group** | **Mean** | **SD** | **Median** | **Q1** | **Q3** | **Min** | **Max** | **N** |
| --- | --- | --- | --- | --- | --- | --- | --- | --- | --- |
| **Baseline** | Placebo | 23.91 | 19.7 | 23.3 | 8.35 | 33.45 | 0.66 | 64.8 | 15 |
| **Baseline** | HDM-SLIT | 74.7 | 175.98 | 22.9 | 7.09 | 31.15 | 2.15 | 721 | 18 |
| **4 Months** | Placebo | 41.99 | 47.82 | 25.5 | 14 | 50.25 | 0.89 | 162 | 15 |
| **4 Months** | HDM-SLIT | 99 | 179.44 | 40.3 | 26.72 | 75.07 | 8.34 | 781 | 18 |
| **12 Months** | Placebo | 48.63 | 58.36 | 30.2 | 14.95 | 61.9 | 0.74 | 222 | 14 |
| **12 Months** | HDM-SLIT | 84.82 | 149.26 | 38.65 | 21.38 | 77.48 | 3.93 | 654 | 18 |
| **24 Months** | Placebo | 81.23 | 120.52 | 35.8 | 18.2 | 70.9 | 0.4 | 447 | 13 |
| **24 Months** | HDM-SLIT | 41.98 | 63.69 | 30.5 | 14.05 | 40.6 | 1.01 | 274 | 16 |

Table e.ii.ii: sIgE Der f extract: Median Regression for change with time trend.

| **Subgroup** | **Comparison** | **Effect** | **Std Error** | **lower CL** | **upper CL** | **p-value** |
| --- | --- | --- | --- | --- | --- | --- |
| **Within Placebo** | 4 M vs. BL | 15.96 | 8.10 | -0.31 | 32.24 | 0.054 |
|  | 12 M vs. BL | 17.86 | 12.13 | -6.52 | 42.24 | 0.1 |
|  | 24 M vs. BL | 47.75 | 21.76 | 4.02 | 91.48 | 0.033 |
|  | 12 M vs. 4 M | 2.68 | 8.25 | -13.91 | 19.26 | 0.7 |
|  | 24 M vs. 4 M | 19.25 | 17.47 | -15.85 | 54.35 | 0.2 |
|  | 24 M vs. 12 M | 13.72 | 12.90 | -12.21 | 39.65 | 0.2 |
| **Within HDM-SLIT** | 4 M vs. BL | 16.50 | 38.32 | -60.50 | 93.50 | 0.6 |
|  | 12 M vs. BL | 11.43 | 41.66 | -72.29 | 95.14 | 0.7 |
|  | 24 M vs. BL | -5.46 | 35.74 | -77.29 | 66.37 | 0.8 |
|  | 12 M vs. 4 M | -11.42 | 13.59 | -38.74 | 15.90 | 0.4 |
|  | 24 M vs. 4 M | -22.88 | 22.21 | -67.52 | 21.76 | 0.3 |
|  | 24 M vs. 12 M | -17.53 | 17.65 | -53.00 | 17.95 | 0.3 |

Table e.i.iii: sIgE Der f extract: Median Regression for difference in change with time trend between groups.

| **Variable** | **Comparison** | **Effect** | **Std Error** | **lower CL** | **upper CL** | **p-value** |
| --- | --- | --- | --- | --- | --- | --- |
| **IA: Time*RD_Group** | 4 M vs. BL | -8.30 | 52.28 | -113.37 | 96.76 | 0.8 |
| **(HDM-SLIT vs. Placebo)** | 12 M vs. BL | -16.26 | 51.34 | -119.43 | 86.92 | 0.7 |
|  | 24 M vs. BL | -57.15 | 57.10 | -171.90 | 57.61 | 0.3 |

## iii. **Der p 1**

Table e.iii.i: sIgE: Der p 1.

| **Time** | **Group** | **Mean** | **SD** | **Median** | **Q1** | **Q3** | **Min** | **Max** | **N** |
| --- | --- | --- | --- | --- | --- | --- | --- | --- | --- |
| **Baseline** | Placebo | 29.94 | 27.1 | 25.83 | 5 | 43.47 | 0 | 91.8 | 15 |
| **Baseline** | HDM-SLIT | 26.72 | 26.5 | 15.78 | 8.71 | 39.47 | 0 | 85.24 | 18 |
| **4 Months** | Placebo | 37.21 | 36.03 | 32.55 | 5.39 | 57.09 | 0 | 103.27 | 15 |
| **4 Months** | HDM-SLIT | 38.58 | 36.14 | 30.44 | 7.76 | 51.99 | 0 | 106.51 | 18 |
| **12 Months** | Placebo | 38.84 | 38.3 | 41.18 | 0.3 | 66.92 | 0 | 105.3 | 13 |
| **12 Months** | HDM-SLIT | 32.68 | 36.56 | 15.05 | 6.95 | 51.92 | 0 | 108.11 | 17 |
| **24 Months** | Placebo | 47.42 | 36.13 | 46.33 | 17.9 | 66.91 | 0 | 106.44 | 13 |
| **24 Months** | HDM-SLIT | 23.63 | 29.34 | 14.97 | 2.78 | 30.95 | 0.15 | 100.32 | 16 |

Table e.ii.ii: sIgE Der p 1: Linear Regression for change with time trend.

| **Subgroup** | **Comparison** | **Effect** | **Std Error** | **lower CL** | **upper CL** | **p-value** |
| --- | --- | --- | --- | --- | --- | --- |
| **Within Placebo** | 4 M vs. BL | 7.26 | 4.80 | -2.00 | 16.53 | 0.1 |
|  | 12 M vs. BL | 6.78 | 5.05 | -2.93 | 16.56 | 0.2 |
|  | 24 M vs. BL | 13.25 | 5.04 | 3.62 | 23.12 | 0.012 |
|  | 12 M vs. 4 M | -0.49 | 5.05 | -10.19 | 9.30 | 0.9 |
|  | 24 M vs. 4 M | 5.99 | 5.04 | -3.64 | 15.85 | 0.2 |
|  | 24 M vs. 12 M | 6.48 | 5.22 | -3.53 | 16.63 | 0.2 |
| **Within HDM-SLIT** | 4 M vs. BL | 11.86 | 4.38 | 3.37 | 20.35 | 0.009 |
|  | 12 M vs. BL | 6.50 | 4.46 | -2.15 | 15.15 | 0.15 |
|  | 24 M vs. BL | 1.53 | 4.55 | -7.40 | 10.27 | 0.7 |
|  | 12 M vs. 4 M | -5.36 | 4.46 | -14.00 | 3.29 | 0.2 |
|  | 24 M vs. 4 M | -10.33 | 4.55 | -19.26 | -1.59 | 0.028 |
|  | 24 M vs. 12 M | -4.97 | 4.64 | -14.07 | 3.93 | 0.29 |

Table e.ii.iii: sIgE Der p 1: Linear Regression for difference in change with time trend between groups.

| **Variable** | **Comparison** | **Effect** | **Std Error** | **lower CL** | **upper CL** | **p-value** |
| --- | --- | --- | --- | --- | --- | --- |
| **IA: Time*RD_Group** | 4 M vs. BL | 4.60 | 6.49 | -7.84 | 17.03 | 0.5 |
| **(HDM-SLIT vs. Placebo)** | 12 M vs. BL | -0.38 | 6.73 | -13.30 | 12.49 | 0.95 |
|  | 24 M vs. BL | -11.64 | 6.79 | -24.73 | 1.30 | 0.09 |

## iv. **Der f 1**

Table e.iv.i: sIgE: Der f 1 (µg/ml).

| **Time** | **Group** | **Mean** | **SD** | **Median** | **Q1** | **Q3** | **Min** | **Max** | **N** |
| --- | --- | --- | --- | --- | --- | --- | --- | --- | --- |
| **Baseline** | Placebo | 18.2 | 16.4 | 15.57 | 3.42 | 30.32 | 0 | 47.09 | 15 |
| **Baseline** | HDM-SLIT | 14.63 | 14.48 | 8.18 | 2.83 | 23.99 | 0 | 47.16 | 18 |
| **4 Months** | Placebo | 28.6 | 29.3 | 20.39 | 2.86 | 45.11 | 0 | 91.96 | 15 |
| **4 Months** | HDM-SLIT | 26.25 | 24.14 | 19.5 | 12.84 | 30.99 | 0 | 96.69 | 18 |
| **12 Months** | Placebo | 31.4 | 35.17 | 19.07 | 0.37 | 49.18 | 0 | 104.23 | 13 |
| **12 Months** | HDM-SLIT | 22.61 | 26.42 | 14.04 | 10.27 | 26.2 | 0 | 103.08 | 17 |
| **24 Months** | Placebo | 38.3 | 37.12 | 20.69 | 13.9 | 55.96 | 0 | 103.46 | 13 |
| **24 Months** | HDM-SLIT | 17.41 | 20.35 | 13.58 | 4.87 | 24.07 | 0 | 85.52 | 16 |

Table e.iv.ii: sIgE Der f 1: Linear Regression for change with time trend.

| **Subgroup** | **Comparison** | **Effect** | **Std Error** | **lower CL** | **upper CL** | **p-value** |
| --- | --- | --- | --- | --- | --- | --- |
| **Within Placebo** | 4 M vs. BL | 10.39 | 5.14 | 0.47 | 20.32 | 0.05 |
|  | 12 M vs. BL | 13.21 | 5.41 | 2.84 | 23.71 | 0.019 |
|  | 24 M vs. BL | 17.90 | 5.40 | 7.57 | 28.44 | 0.002 |
|  | 12 M vs. 4 M | 2.81 | 5.41 | -7.56 | 13.32 | 0.6 |
|  | 24 M vs. 4 M | 7.50 | 5.40 | -2.82 | 18.05 | 0.2 |
|  | 24 M vs. 12 M | 4.69 | 5.59 | -6.06 | 15.52 | 0.4 |
| **Within HDM-SLIT** | 4 M vs. BL | 11.62 | 4.58 | 2.75 | 20.49 | 0.014 |
|  | 12 M vs. BL | 7.82 | 4.66 | -1.18 | 16.88 | 0.1 |
|  | 24 M vs. BL | 4.57 | 4.76 | -4.75 | 13.69 | 0.3 |
|  | 12 M vs. 4 M | -3.80 | 4.66 | -12.81 | 5.26 | 0.4 |
|  | 24 M vs. 4 M | -7.05 | 4.76 | -16.37 | 2.06 | 0.1 |

Table e.iv.iii: sIgE Der f 1: Linear Regression for difference in change with time trend between groups.

| **Variable** | **Comparison** | **Effect** | **Std Error** | **lower CL** | **upper CL** | **p-value** |
| --- | --- | --- | --- | --- | --- | --- |
| **IA: Time*RD_Group** | 4 M vs. BL | 1.23 | 6.86 | -11.91 | 14.36 | 0.85 |
| **(HDM-SLIT vs. Placebo)** | 12 M vs. BL | -5.39 | 7.11 | -19.02 | 8.20 | 0.45 |
|  | 24 M vs. BL | -13.28 | 7.17 | -27.11 | 0.37 | 0.07 |

## v. **Der p 2**

Table e.v.i: sIgE: Der p 2 (µg/ml).

| **Time** | **Group** | **Mean** | **SD** | **Median** | **Q1** | **Q3** | **Min** | **Max** | **N** |
| --- | --- | --- | --- | --- | --- | --- | --- | --- | --- |
| **Baseline** | Placebo | 16.31 | 19.42 | 10.23 | 0.54 | 25.33 | 0 | 58.81 | 15 |
| **Baseline** | HDM-SLIT | 39.68 | 34.6 | 33.88 | 10.83 | 56.89 | 0 | 105.1 | 18 |
| **4 Months** | Placebo | 29.77 | 33.2 | 22.93 | 1.19 | 45.35 | 0 | 101.8 | 15 |
| **4 Months** | HDM-SLIT | 39.36 | 34.42 | 28.7 | 14.52 | 50.66 | 0.33 | 105.23 | 18 |
| **12 Months** | Placebo | 35.71 | 36.94 | 34.78 | 0.46 | 69.66 | 0 | 103.89 | 13 |
| **12 Months** | HDM-SLIT | 34.63 | 30.33 | 29.66 | 8.81 | 51.65 | 1.02 | 108.19 | 17 |
| **24 Months** | Placebo | 47.53 | 39.91 | 50.36 | 1.23 | 82.43 | 0 | 104.97 | 13 |
| **24 Months** | HDM-SLIT | 26.49 | 26.23 | 17.39 | 13.84 | 30.84 | 0.57 | 104.6 | 16 |

Table e.v.ii: sIgE Der p 2: Linear Regression for change with time trend.

| **Subgroup** | **Comparison** | **Effect** | **Std Error** | **lower CL** | **upper CL** | **p-value** |
| --- | --- | --- | --- | --- | --- | --- |
| **Within Placebo** | 4 M vs. BL | 13.45 | 6.86 | 0.21 | 26.70 | 0.057 |
|  | 12 M vs. BL | 21.14 | 7.21 | 7.28 | 35.07 | 0.006 |
|  | 24 M vs. BL | 30.79 | 7.20 | 17.05 | 44.82 | <0.001 |
|  | 12 M vs. 4 M | 7.69 | 7.21 | -6.17 | 21.62 | 0.3 |
|  | 24 M vs. 4 M | 17.34 | 7.20 | 3.59 | 31.37 | 0.021 |
|  | 24 M vs. 12 M | 9.65 | 7.45 | -4.62 | 24.13 | 0.2 |
| **Within** **HDM-SLIT** | 4 M vs. BL | -0.32 | 5.40 | -10.79 | 10.15 | 0.95 |
|  | 12 M vs. BL | -4.60 | 5.50 | -15.24 | 6.09 | 0.4 |
|  | 24 M vs. BL | -10.41 | 5.62 | -21.42 | 0.36 | 0.07 |
|  | 12 M vs. 4 M | -4.28 | 5.50 | -14.92 | 6.41 | 0.4 |
|  | 24 M vs. 4 M | -10.09 | 5.62 | -21.10 | 0.67 | 0.08 |
|  | 24 M vs. 12 M | -5.81 | 5.72 | -17.05 | 5.13 | 0.3 |

Table e.v.iii: sIgE Der p 2: Linear Regression for difference in change with time trend between groups.

| **Variable** | **Comparison** | **Effect** | **Std Error** | **lower CL** | **upper CL** | **p-value** |
| --- | --- | --- | --- | --- | --- | --- |
| **IA: Time*RD_Group** | 4 M vs. BL | -13.77 | 8.61 | -30.26 | 2.72 | 0.113 |
| **(HDM-SLIT vs. Placebo)** | 12 M vs. BL | -25.51 | 8.92 | -42.57 | -8.40 | 0.005 |
|  | 24 M vs. BL | -40.79 | 9.00 | -58.09 | -23.61 | <0.001 |

## vi. **Der f 2**

Table e.vi.i: sIgE: Der f 2 (µg/ml).

| **Time** | **Group** | **Mean** | **SD** | **Median** | **Q1** | **Q3** | **Min** | **Max** | **N** |
| --- | --- | --- | --- | --- | --- | --- | --- | --- | --- |
| **Baseline** | Placebo | 18.92 | 23.46 | 12.28 | 0.73 | 28.74 | 0 | 83.79 | 15 |
| **Baseline** | HDM-SLIT | 38.05 | 33.79 | 33.36 | 10.31 | 57.94 | 0 | 106.05 | 18 |
| **4 Months** | Placebo | 33.26 | 34.79 | 26.79 | 1.77 | 58.67 | 0 | 105 | 15 |
| **4 Months** | HDM-SLIT | 42.35 | 35.19 | 28.15 | 17.63 | 55.91 | 0.33 | 107.09 | 18 |
| **12 Months** | Placebo | 39.72 | 42.16 | 21.27 | 0.59 | 78.57 | 0 | 104.9 | 13 |
| **12 Months** | HDM-SLIT | 37.07 | 31.11 | 32.36 | 9.96 | 58.62 | 1.94 | 108.21 | 17 |
| **24 Months** | Placebo | 46.92 | 38.52 | 62.2 | 1.09 | 69.73 | 0 | 104.78 | 13 |
| **24 Months** | HDM-SLIT | 27.48 | 26.4 | 19.1 | 13.11 | 32.77 | 0.83 | 105.94 | 16 |

Table e.vi.ii: sIgE Der f 2: Linear Regression for change with time trend.

| **Subgroup** | **Comparison** | **Effect** | **Std Error** | **lower CL** | **upper CL** | **p-value** |
| --- | --- | --- | --- | --- | --- | --- |
| **Within Placebo** | 4 M vs. BL | 14.34 | 6.58 | 1.64 | 27.04 | 0.036 |
|  | 12 M vs. BL | 23.62 | 6.91 | 10.30 | 36.96 | 0.002 |
|  | 24 M vs. BL | 28.19 | 6.91 | 14.93 | 41.57 | <0.001 |
|  | 12 M vs. 4 M | 9.28 | 6.91 | -4.04 | 22.61 | 0.2 |
|  | 24 M vs. 4 M | 13.85 | 6.91 | 0.59 | 27.23 | 0.052 |
|  | 24 M vs. 12 M | 4.57 | 7.15 | -9.16 | 18.41 | 0.5 |
| **Within HDM-SLIT** | 4 M vs. BL | 4.30 | 6.03 | -7.39 | 16.00 | 0.5 |
|  | 12 M vs. BL | -0.81 | 6.15 | -12.68 | 11.15 | 0.9 |
|  | 24 M vs. BL | -7.88 | 6.27 | -20.19 | 4.12 | 0.2 |
|  | 12 M vs. 4 M | -5.11 | 6.15 | -16.98 | 6.85 | 0.4 |
|  | 24 M vs. 4 M | -12.19 | 6.27 | -24.50 | -0.18 | 0.058 |
|  | 24 M vs. 12 M | -7.07 | 6.39 | -19.66 | 5.12 | 0.3 |

Table e.vi.iii: sIgE Der f 2: Linear Regression for difference in change with time trend between groups.

| **Variable** | **Comparison** | **Effect** | **Std Error** | **lower CL** | **upper CL** | **p-value** |
| --- | --- | --- | --- | --- | --- | --- |
| **IA: Time*RD_Group** | 4 M vs. BL | -10.04 | 8.94 | -27.16 | 7.09 | 0.3 |
| **(HDM-SLIT vs. Placebo)** | 12 M vs. BL | -24.18 | 9.27 | -41.87 | -6.38 | 0.011 |
|  | 24 M vs. BL | -35.68 | 9.35 | -53.63 | -17.82 | <0.001 |

## vii. **Der p 23**

Table e.vii.i: sIgE: Der p 23 (µg/ml).

| **Time** | **Group** | **Mean** | **SD** | **Median** | **Q1** | **Q3** | **Min** | **Max** | **N** |
| --- | --- | --- | --- | --- | --- | --- | --- | --- | --- |
| **Baseline** | Placebo | 4.65 | 11.23 | 0 | 0 | 1.48 | 0 | 42.11 | 15 |
| **Baseline** | HDM-SLIT | 13.62 | 24.41 | 4.38 | 0 | 15.53 | 0 | 100.38 | 18 |
| **4 Months** | Placebo | 5.62 | 10.61 | 0 | 0 | 3.96 | 0 | 30.54 | 15 |
| **4 Months** | HDM-SLIT | 24.49 | 30.05 | 11.82 | 0.82 | 42.59 | 0 | 91.09 | 18 |
| **12 Months** | Placebo | 8.3 | 14.58 | 0 | 0 | 4.47 | 0 | 37.32 | 13 |
| **12 Months** | HDM-SLIT | 21.61 | 21.69 | 13.68 | 0.36 | 39.18 | 0 | 56.99 | 17 |
| **24 Months** | Placebo | 18.36 | 26.85 | 0 | 0 | 29.01 | 0 | 76.18 | 13 |
| **24 Months** | HDM-SLIT | 10.15 | 16.47 | 5.14 | 0.22 | 10.5 | 0 | 61.68 | 16 |

Table e.vii.ii: sIgE Der p 23: Median Regression for change with time trend.

| **Subgroup** | **Comparison** | **Effect** | **Std Error** | **lower CL** | **upper CL** | **p-value** |
| --- | --- | --- | --- | --- | --- | --- |
| **Within Placebo** | 4 M vs. BL | 1.22 | 1.55 | -1.89 | 4.34 | 0.4 |
|  | 12 M vs. BL | 2.77 | 2.86 | -2.98 | 8.52 | 0.3 |
|  | 24 M vs. BL | 11.68 | 6.89 | -2.17 | 25.52 | 0.1 |
|  | 12 M vs. 4 M | 2.11 | 2.55 | -3.01 | 7.23 | 0.4 |
|  | 24 M vs. 4 M | 11.31 | 6.83 | -2.42 | 25.04 | 0.1 |
|  | 24 M vs. 12 M | 7.18 | 5.12 | -3.11 | 17.48 | 0.2 |
| **Within HDM-SLIT** | 4 M vs. BL | 7.91 | 5.02 | -2.17 | 18.00 | 0.1 |
|  | 12 M vs. BL | 5.94 | 4.15 | -2.41 | 14.28 | 0.15 |
|  | 24 M vs. BL | -0.75 | 4.47 | -9.73 | 8.23 | 0.9 |
|  | 12 M vs. 4 M | -4.30 | 3.87 | -12.07 | 3.47 | 0.3 |
|  | 24 M vs. 4 M | -11.53 | 5.08 | -21.74 | -1.32 | 0.028 |
|  | 24 M vs. 12 M | -9.50 | 4.48 | -18.50 | -0.49 | 0.039 |

Table e.vii.iii: sIgE Der p 23: Median Regression for difference in change with time trend between groups.

| **Variable** | **Comparison** | **Effect** | **Std Error** | **lower CL** | **upper CL** | **p-value** |
| --- | --- | --- | --- | --- | --- | --- |
| **IA: Time*RD_Group** | 4 M vs. BL | 7.80 | 6.09 | -4.44 | 20.03 | 0.2 |
| **(HDM-SLIT vs. Placebo)** | 12 M vs. BL | 4.02 | 5.81 | -7.65 | 15.69 | 0.4 |
|  | 24 M vs. BL | -15.35 | 7.83 | -31.09 | 0.40 | 0.056 |

## f) **Skin Prick Test**

## i. **Der p-extract_Undiluted**

Table f.i.i: Skin Wheal Size: Der p-extract Undiluted (mm).

| **Time** | **Group** | **Mean** | **SD** | **Median** | **Q1** | **Q3** | **Min** | **Max** | **N** |
| --- | --- | --- | --- | --- | --- | --- | --- | --- | --- |
| **Baseline** | Placebo | 6.67 | 3.93 | 5 | 3 | 9 | 2 | 14 | 15 |
| **Baseline** | HDM-SLIT | 7.14 | 3.09 | 7 | 5 | 7.88 | 2.5 | 14 | 18 |
| **4 Months** | Placebo | 6.5 | 4.9 | 6 | 3.25 | 8 | 0 | 21 | 14 |
| **4 Months** | HDM-SLIT | 5.39 | 2.33 | 5 | 3.25 | 7 | 2 | 10 | 18 |
| **12 Months** | Placebo | 5.29 | 4.07 | 5 | 2.25 | 6.5 | 0 | 13.5 | 14 |
| **12 Months** | HDM-SLIT | 4.44 | 2.52 | 4 | 3.12 | 5 | 0 | 9 | 18 |
| **24 Months** | Placebo | 4.92 | 3.42 | 5 | 2.75 | 7.25 | 0 | 10 | 12 |
| **24 Months** | HDM-SLIT | 5.27 | 3.37 | 4 | 3 | 6 | 3 | 15 | 15 |

Table f.i.ii: Der p-extract Undiluted: Linear Regression for change with time trend.

| **Subgroup** | **Comparison** | **Effect** | **Std Error** | **lower CL** | **upper CL** | **p-value** |
| --- | --- | --- | --- | --- | --- | --- |
| **Within Placebo** | 4 M vs. BL | -0.24 | 1.03 | -2.20 | 1.76 | 0.8 |
|  | 12 M vs. BL | -1.33 | 1.03 | -3.31 | 0.65 | 0.2 |
|  | 24 M vs. BL | -2.06 | 1.08 | -4.10 | 0.07 | 0.064 |
|  | 12 M vs. 4 M | -1.09 | 1.05 | -3.13 | 0.91 | 0.3 |
|  | 24 M vs. 4 M | -1.82 | 1.09 | -3.90 | 0.30 | 0.1 |
|  | 24 M vs. 12 M | -0.73 | 1.09 | -2.79 | 1.42 | 0.5 |
| **Within HDM-SLIT** | 4 M vs. BL | -1.75 | 0.85 | -3.40 | -0.10 | 0.045 |
|  | 12 M vs. BL | -2.69 | 0.85 | -4.34 | -1.05 | 0.003 |
|  | 24 M vs. BL | -1.92 | 0.90 | -3.65 | -0.18 | 0.038 |
|  | 12 M vs. 4 M | -0.94 | 0.85 | -2.59 | 0.70 | 0.2 |
|  | 24 M vs. 4 M | -0.17 | 0.90 | -1.90 | 1.57 | 0.8 |
|  | 24 M vs. 12 M | 0.78 | 0.90 | -0.96 | 2.51 | 0.3 |

Table f.i.iii: Der p-extract Undiluted: Linear Regression for difference in change with time trend between groups.

| **Variable** | **Comparison** | **Effect** | **Std Error** | **lower CL** | **upper CL** | **p-value** |
| --- | --- | --- | --- | --- | --- | --- |
| **IA: Time*RD_Group** | 4 M vs. BL | -1.53 | 1.32 | -4.06 | 0.99 | 0.2 |
| **(HDM-SLIT vs. Placebo)** | 12 M vs. BL | -1.37 | 1.32 | -3.90 | 1.16 | 0.3 |
|  | 24 M vs. BL | 0.08 | 1.39 | -2.61 | 2.73 | 0.9 |

## ii. **Der p-extract_1:2 diluted**

Table f.ii.i: Skin Wheal Size: Der p-extract 1:2 diluted.

| **Time** | **Group** | **Mean** | **SD** | **Median** | **Q1** | **Q3** | **Min** | **Max** | **N** |
| --- | --- | --- | --- | --- | --- | --- | --- | --- | --- |
| **Baseline** | Placebo | 7.03 | 3.44 | 7 | 5 | 8.75 | 0 | 14 | 15 |
| **Baseline** | HDM-SLIT | 7.5 | 4.57 | 6.25 | 4.62 | 8.75 | 3 | 21 | 18 |
| **4 Months** | Placebo | 6.68 | 3.89 | 5.5 | 5 | 8.25 | 0 | 13 | 14 |
| **4 Months** | HDM-SLIT | 5.31 | 2.85 | 4.5 | 4 | 6.5 | 0 | 12 | 18 |
| **12 Months** | Placebo | 6.43 | 4.84 | 5.25 | 3 | 9.75 | 0 | 15 | 14 |
| **12 Months** | HDM-SLIT | 4.03 | 3.12 | 3 | 2 | 4.88 | 0 | 12.5 | 18 |
| **24 Months** | Placebo | 4.18 | 1.81 | 5 | 3.75 | 5 | 0 | 6.5 | 11 |
| **24 Months** | HDM-SLIT | 3.67 | 1.76 | 3 | 2 | 4.5 | 2 | 8 | 15 |

Table f.ii.ii: Der p-extract 1:2 diluted: Linear Regression for change with time trend.

| **Subgroup** | **Comparison** | **Effect** | **Std Error** | **lower CL** | **upper CL** | **p-value** |
| --- | --- | --- | --- | --- | --- | --- |
| **Within Placebo** | 4 M vs. BL | -0.34 | 0.84 | -1.96 | 1.28 | 0.6 |
|  | 12 M vs. BL | -0.57 | 0.84 | -2.19 | 1.05 | 0.5 |
|  | 24 M vs. BL | -3.14 | 0.91 | -4.89 | -1.37 | 0.001 |
|  | 12 M vs. 4 M | -0.23 | 0.86 | -1.89 | 1.43 | 0.7 |
|  | 24 M vs. 4 M | -2.80 | 0.92 | -4.57 | -1.02 | 0.004 |
|  | 24 M vs. 12 M | -2.57 | 0.92 | -4.34 | -0.79 | 0.008 |
| **Within HDM-SLIT** | 4 M vs. BL | -2.19 | 0.90 | -3.93 | -0.46 | 0.018 |
|  | 12 M vs. BL | -3.47 | 0.90 | -5.21 | -1.74 | <0.001 |
|  | 24 M vs. BL | -4.08 | 0.95 | -5.89 | -2.21 | <0.001 |
|  | 12 M vs. 4 M | -1.28 | 0.90 | -3.01 | 0.46 | 0.1 |
|  | 24 M vs. 4 M | -1.88 | 0.95 | -3.69 | -0.02 | 0.052 |
|  | 24 M vs. 12 M | -0.61 | 0.95 | -2.41 | 1.26 | 0.5 |

Table f.ii.iii: Skin Der p-extract 1:2: Linear Regression for difference in change with time trend between groups.

| **Variable** | **Comparison** | **Effect** | **Std Error** | **lower CL** | **upper CL** | **p-value** |
| --- | --- | --- | --- | --- | --- | --- |
| **IA: Time*RD_Group** | 4 M vs. BL | -1.86 | 1.25 | -4.26 | 0.54 | 0.1 |
| **(HDM-SLIT vs. Placebo)** | 12 M vs. BL | -2.90 | 1.25 | -5.30 | -0.50 | 0.023 |
|  | 24 M vs. BL | -1.03 | 1.35 | -3.61 | 1.54 | 0.4 |

## iii. **Der p-extract_1:5 diluted**

Table f.iii.i: Skin Wheal Size: Der p-extract 1:5 diluted.

| **Time** | **Group** | **Mean** | **SD** | **Median** | **Q1** | **Q3** | **Min** | **Max** | **N** |
| --- | --- | --- | --- | --- | --- | --- | --- | --- | --- |
| **Baseline** | Placebo | 5.6 | 3.54 | 4 | 3 | 8.25 | 0 | 13 | 15 |
| **Baseline** | HDM-SLIT | 5.53 | 3.64 | 4.5 | 3 | 8.12 | 0 | 13 | 18 |
| **4 Months** | Placebo | 5.93 | 3.78 | 6 | 4.12 | 7.88 | 0 | 12.5 | 14 |
| **4 Months** | HDM-SLIT | 3.78 | 1.94 | 3.5 | 3 | 4 | 0 | 8.5 | 18 |
| **12 Months** | Placebo | 5.29 | 5.31 | 3.5 | 1.5 | 7.5 | 0 | 18 | 14 |
| **12 Months** | HDM-SLIT | 3.22 | 2.05 | 3 | 2 | 4 | 0 | 8 | 18 |
| **24 Months** | Placebo | 3.45 | 2.11 | 3.5 | 2 | 4.75 | 0 | 6.5 | 11 |
| **24 Months** | HDM-SLIT | 3 | 2.9 | 2 | 1.5 | 4 | 0 | 11.5 | 15 |

Table f.iii.ii: Skin Der p-extract 1:5 diluted: Linear Regression for change with time trend.

| **Subgroup** | **Comparison** | **Effect** | **Std Error** | **lower CL** | **upper CL** | **p-value** |
| --- | --- | --- | --- | --- | --- | --- |
| **Within Placebo** | 4 M vs. BL | 0.22 | 0.91 | -1.51 | 1.98 | 0.8 |
|  | 12 M vs. BL | -0.20 | 0.91 | -1.96 | 1.54 | 0.8 |
|  | 24 M vs. BL | -2.62 | 0.99 | -4.50 | -0.69 | 0.012 |
|  | 12 M vs. 4 M | -0.42 | 0.93 | -2.24 | 1.34 | 0.6 |
|  | 24 M vs. 4 M | -2.84 | 1.00 | -4.75 | -0.91 | 0.007 |
|  | 24 M vs. 12 M | -2.41 | 1.00 | -4.30 | -0.46 | 0.02 |
| **Within HDM-SLIT** | 4 M vs. BL | -1.75 | 0.81 | -3.31 | -0.19 | 0.035 |
|  | 12 M vs. BL | -2.31 | 0.81 | -3.87 | -0.74 | 0.006 |
|  | 24 M vs. BL | -2.58 | 0.85 | -4.22 | -0.93 | 0.004 |
|  | 12 M vs. 4 M | -0.56 | 0.81 | -2.12 | 1.01 | 0.4 |
|  | 24 M vs. 4 M | -0.83 | 0.85 | -2.47 | 0.82 | 0.3 |
|  | 24 M vs. 12 M | -0.27 | 0.85 | -1.92 | 1.38 | 0.7 |

Table f.iii.iii: Skin Der p-extract 1:5 diluted: Linear Regression for difference in change with time trend between groups.

| **Variable** | **Comparison** | **Effect** | **Std Error** | **lower CL** | **upper CL** | **p-value** |
| --- | --- | --- | --- | --- | --- | --- |
| **IA: Time*RD_Group** | 4 M vs. BL | -2.00 | 1.21 | -4.33 | 0.31 | 0.1 |
| **(HDM-SLIT vs. Placebo)** | 12 M vs. BL | -2.09 | 1.21 | -4.40 | 0.24 | 0.088 |
|  | 24 M vs. BL | -0.07 | 1.30 | -2.58 | 2.40 | 0.9 |

## iv. **Der p-extract_1:10 diluted**

Table f.iv.i: Skin Wheal Size: Der p-extract 1:10 diluted.

| **Time** | **Group** | **Mean** | **SD** | **Median** | **Q1** | **Q3** | **Min** | **Max** | **N** |
| --- | --- | --- | --- | --- | --- | --- | --- | --- | --- |
| **Baseline** | Placebo | 3.63 | 3 | 3 | 1.5 | 5 | 0 | 11 | 15 |
| **Baseline** | HDM-SLIT | 4.58 | 3.8 | 3.25 | 2.25 | 6.5 | 0 | 15 | 18 |
| **4 Months** | Placebo | 4.57 | 3.69 | 4 | 2 | 6.5 | 0 | 12.5 | 14 |
| **4 Months** | HDM-SLIT | 2.69 | 2.37 | 3 | 2 | 3 | 0 | 10 | 18 |
| **12 Months** | Placebo | 3.14 | 2.63 | 3 | 0.5 | 5 | 0 | 8 | 14 |
| **12 Months** | HDM-SLIT | 2.36 | 2.24 | 2 | 1 | 3.62 | 0 | 8 | 18 |
| **24 Months** | Placebo | 2.73 | 2.38 | 3 | 0.5 | 4 | 0 | 7.5 | 11 |
| **24 Months** | HDM-SLIT | 1.47 | 1.6 | 1 | 0 | 2 | 0 | 5 | 15 |

Table f.iv.ii: Skin Der p-extract 1:10 diluted: Linear Regression for change with time trend.

| **Subgroup** | **Comparison** | **Effect** | **Std Error** | **lower CL** | **upper CL** | **p-value** |
| --- | --- | --- | --- | --- | --- | --- |
| **Within Placebo** | 4 M vs. BL | 0.90 | 0.67 | -0.39 | 2.20 | 0.1 |
|  | 12 M vs. BL | -0.36 | 0.67 | -1.67 | 0.93 | 0.5 |
|  | 24 M vs. BL | -1.10 | 0.73 | -2.51 | 0.31 | 0.1 |
|  | 12 M vs. 4 M | -1.25 | 0.69 | -2.61 | 0.05 | 0.077 |
|  | 24 M vs. 4 M | -2.00 | 0.74 | -3.43 | -0.58 | 0.01 |
|  | 24 M vs. 12 M | -0.74 | 0.74 | -2.15 | 0.70 | 0.3 |
| **Within HDM-SLIT** | 4 M vs. BL | -1.89 | 0.75 | -3.35 | -0.43 | 0.016 |
|  | 12 M vs. BL | -2.22 | 0.75 | -3.68 | -0.76 | 0.005 |
|  | 24 M vs. BL | -3.12 | 0.80 | -4.67 | -1.58 | <0.001 |
|  | 12 M vs. 4 M | -0.33 | 0.75 | -1.79 | 1.13 | 0.6 |
|  | 24 M vs. 4 M | -1.23 | 0.80 | -2.78 | 0.31 | 0.1 |
|  | 24 M vs. 12 M | -0.89 | 0.80 | -2.44 | 0.64 | 0.2 |

Table f.iv.iii: Skin Der p-extract 1:10 diluted: Linear Regression for difference in change with time trend between groups.

| **Variable** | **Comparison** | **Effect** | **Std Error** | **lower CL** | **upper CL** | **p-value** |
| --- | --- | --- | --- | --- | --- | --- |
| **IA: Time*RD_Group** | 4 M vs. BL | -2.80 | 1.04 | -4.80 | -0.82 | 0.009 |
| **(HDM-SLIT vs. Placebo)** | 12 M vs. BL | -1.85 | 1.04 | -3.83 | 0.15 | 0.079 |
|  | 24 M vs. BL | -2.08 | 1.12 | -4.23 | 0.04 | 0.065 |

## g) **BAT**

## i. **Half-maximum eliciting concentration (EC_50_) of Der p-extract**

Table g.i.i: BAT: EC_50_.

| **Time** | **Group** | **Mean** | **SD** | **Median** | **Q1** | **Q3** | **Min** | **Max** | **N** |
| --- | --- | --- | --- | --- | --- | --- | --- | --- | --- |
| **Baseline** | Placebo | 66.54 | 108.01 | 10.3 | 2.78 | 59.3 | 1.71 | 278 | 12 |
| **Baseline** | HDM-SLIT | 36.22 | 68.69 | 1.22 | 0.69 | 21.5 | 0 | 225 | 17 |
| **4 Months** | Placebo | 32.83 | 65.02 | 5.61 | 2.94 | 10.13 | 1.56 | 195 | 12 |
| **4 Months** | HDM-SLIT | 8 | 9.57 | 5.33 | 0.83 | 8.39 | 0.18 | 27 | 16 |
| **12 Months** | Placebo | 7.75 | 11.28 | 2.03 | 1.42 | 6.96 | 0.02 | 29.5 | 10 |
| **12 Months** | HDM-SLIT | 57.46 | 113.49 | 17.5 | 4.73 | 36.5 | 0.22 | 411.7 | 15 |
| **24 Months** | Placebo | 28.82 | 73.75 | 5.04 | 2.09 | 8.05 | 0.14 | 225.3 | 9 |
| **24 Months** | HDM-SLIT | 76.77 | 104.1 | 28.54 | 21.18 | 45.53 | 7.27 | 285 | 14 |

Table g.i.ii: EC50: Median Regression for change with time trend.

| **Subgroup** | **Comparison** | **Effect** | **Std Error** | **lower CL** | **upper CL** | **p-value** |
| --- | --- | --- | --- | --- | --- | --- |
| **Within Placebo** | 4 M vs. BL | -31.53 | 14.59 | -60.85 | -2.22 | 0.036 |
|  | 12 M vs. BL | -47.77 | 24.09 | -96.17 | 0.64 | 0.053 |
|  | 24 M vs. BL | -31.89 | 17.81 | -67.69 | 3.90 | 0.080 |
|  | 12 M vs. 4 M | -19.04 | 13.94 | -47.06 | 8.98 | 0.1 |
|  | 24 M vs. 4 M | -0.93 | 10.18 | -21.40 | 19.53 | 0.9 |
|  | 24 M vs. 12 M | 22.87 | 17.23 | -11.75 | 57.50 | 0.1 |
| **Within HDM-SLIT** | 4 M vs. BL | -3.22 | 14.78 | -32.92 | 26.48 | 0.8 |
|  | 12 M vs. BL | 13.13 | 20.19 | -27.44 | 53.70 | 0.5 |
|  | 24 M vs. BL | 30.47 | 23.78 | -17.32 | 78.26 | 0.2 |
|  | 12 M vs. 4 M | 22.51 | 18.40 | -14.47 | 59.48 | 0.2 |
|  | 24 M vs. 4 M | 38.82 | 21.64 | -4.68 | 82.31 | 0.079 |
|  | 24 M vs. 12 M | 14.08 | 21.12 | -28.37 | 56.52 | 0.5 |

Table g.i.iii: EC_50_: Median Regression for difference for change with time trend between groups.

| **Variable** | **Comparison** | **Effect** | **Std Error** | **lower CL** | **upper CL** | **p-value** |
| --- | --- | --- | --- | --- | --- | --- |
| **IA: Time*RD_Group** | 4 M vs. BL | 23.64 | 24.43 | -25.45 | 72.73 | 0.3 |
| **(HDM-SLIT vs. Placebo)** | 12 M vs. BL | 71.80 | 36.27 | -1.08 | 144.69 | 0.053 |
|  | 24 M vs. BL | 68.86 | 27.56 | 13.48 | 124.25 | 0.016 |

## ii. **Sensitivity and reactivity (AUC) to Der p-extract**

Table g.ii.i: BAT: AUC.

| **Time** | **Group** | **Mean** | **SD** | **Median** | **Q1** | **Q3** | **Min** | **Max** | **N** |
| --- | --- | --- | --- | --- | --- | --- | --- | --- | --- |
| **Baseline** | Placebo | 129.95 | 98.48 | 117.07 | 41.24 | 182.11 | 19.65 | 299.45 | 12 |
| **Baseline** | HDM-SLIT | 238.2 | 140.34 | 216.55 | 118.6 | 359 | 28.7 | 448.4 | 17 |
| **4 Months** | Placebo | 146.98 | 96.09 | 135.2 | 73.8 | 228.95 | 18.45 | 278.14 | 12 |
| **4 Months** | HDM-SLIT | 212.71 | 137.26 | 164.38 | 138.09 | 247.7 | 61 | 543.1 | 16 |
| **12 Months** | Placebo | 181.62 | 104.93 | 182.35 | 136.93 | 257.96 | 15.3 | 326.05 | 10 |
| **12 Months** | HDM-SLIT | 164.78 | 110.16 | 112.85 | 88.57 | 220.65 | 51.6 | 435.1 | 15 |
| **24 Months** | Placebo | 194.56 | 155.86 | 177.22 | 85.52 | 235.98 | 9.1 | 462.9 | 10 |
| **24 Months** | HDM-SLIT | 163.7 | 141.19 | 144.28 | 96.89 | 175.43 | 23.6 | 616.5 | 15 |

Table g.ii.ii: AUC: Linear Regression for change with time trend.

| **Subgroup** | **Comparison** | **Effect** | **Std Error** | **lower CL** | **upper CL** | **p-value** |
| --- | --- | --- | --- | --- | --- | --- |
| **Within Placebo** | 4 M vs. BL | 17.03 | 22.68 | -26.55 | 60.61 | 0.4 |
|  | 12 M vs. BL | 46.54 | 24.25 | 0.11 | 93.18 | 0.065 |
|  | 24 M vs. BL | 59.48 | 24.25 | 13.05 | 106.12 | 0.020 |
|  | 12 M vs. 4 M | 29.51 | 24.25 | -16.92 | 76.15 | 0.2 |
|  | 24 M vs. 4 M | 42.45 | 24.25 | -3.98 | 89.09 | 0.090 |
|  | 24 M vs. 12 M | 12.94 | 24.85 | -34.81 | 60.68 | 0.6 |
| **Within HDM-SLIT** | 4 M vs. BL | -32.64 | 33.00 | -95.79 | 31.88 | 0.3 |
|  | 12 M vs. BL | -82.67 | 33.71 | -146.90 | -16.46 | 0.018 |
|  | 24 M vs. BL | -77.54 | 33.64 | -142.66 | -12.58 | 0.026 |
|  | 12 M vs. 4 M | -50.03 | 34.01 | -115.51 | 16.07 | 0.1 |
|  | 24 M vs. 4 M | -44.90 | 34.33 | -112.00 | 20.69 | 0.1 |
|  | 24 M vs. 12 M | 5.13 | 35.05 | -63.67 | 71.82 | 0.8 |

Table g.ii.iii: AUC: Linear Regression for difference of change with time trend between groups.

| **Variable** | **Comparison** | **Effect** | **Std Error** | **lower CL** | **upper CL** | **p-value** |
| --- | --- | --- | --- | --- | --- | --- |
| **IA: Time*RD_Group** | 4 M vs. BL | -50.26 | 43.53 | -132.95 | 33.02 | 0.2 |
| **(HDM-SLIT vs. Placebo)** | 12 M vs. BL | -130.63 | 45.60 | -217.21 | -43.43 | 0.005 |
|  | 24 M vs. BL | -137.56 | 45.56 | -224.52 | -50.90 | 0.003 |

## h) **Number of Sensitizations to allergen extracts**

## i. **In serum and skin, combined**

Table h.i.i: Number of Sensitizations

.

| **Time** | **Group** | **Mean** | **SD** | **Median** | **Q1** | **Q3** | **Min** | **Max** | **N** |
| --- | --- | --- | --- | --- | --- | --- | --- | --- | --- |
| **Baseline** | Placebo | 2.13 | 1.19 | 2 | 1 | 2.5 | 1 | 5 | 15 |
| **Baseline** | HDM-SLIT | 1.94 | 1.06 | 2 | 1 | 2.75 | 1 | 4 | 18 |
| **4 Months** | Placebo | 2.5 | 1.34 | 2 | 1.25 | 3.75 | 1 | 5 | 14 |
| **4 Months** | HDM-SLIT | 1.72 | 1.18 | 1 | 1 | 2 | 1 | 5 | 18 |
| **12 Months** | Placebo | 3.14 | 1.88 | 3 | 1.25 | 4.75 | 1 | 6 | 14 |
| **12 Months** | HDM-SLIT | 2.39 | 1.38 | 2 | 1 | 3 | 1 | 5 | 18 |
| **24 Months** | Placebo | 3.42 | 2.02 | 3.5 | 1 | 5 | 1 | 6 | 12 |
| **24 Months** | HDM-SLIT | 2 | 1.41 | 1 | 1 | 3 | 1 | 6 | 15 |

Table h.i.ii: Number of Sensitizations: Median Regression for change with time trend.

| **Subgroup** | **Comparison** | **Effect** | **Std Error** | **lower CL** | **upper CL** | **p-value** |
| --- | --- | --- | --- | --- | --- | --- |
| **Within Placebo** | 4 M vs. BL | 0.46 | 0.24 | -0.02 | 0.93 | 0.060 |
|  | 12 M vs. BL | 0.92 | 0.36 | 0.19 | 1.64 | 0.015 |
|  | 24 M vs. BL | 1.20 | 0.46 | 0.27 | 2.13 | 0.013 |
|  | 12 M vs. 4 M | 0.60 | 0.38 | -0.16 | 1.36 | 0.1 |
|  | 24 M vs. 4 M | 0.85 | 0.42 | 0.00 | 1.70 | 0.049 |
|  | 24 M vs. 12 M | 0.22 | 0.31 | -0.40 | 0.83 | 0.4 |
| **Within HDM-SLIT** | 4 M vs. BL | -0.19 | 0.16 | -0.51 | 0.12 | 0.2 |
|  | 12 M vs. BL | 0.44 | 0.25 | -0.07 | 0.95 | 0.092 |
|  | 24 M vs. BL | 0.01 | 0.33 | -0.66 | 0.69 | 0.9 |
|  | 12 M vs. 4 M | 0.66 | 0.19 | 0.27 | 1.04 | 0.001 |
|  | 24 M vs. 4 M | 0.24 | 0.38 | -0.53 | 1.01 | 0.5 |
|  | 24 M vs. 12 M | -0.43 | 0.30 | -1.04 | 0.17 | 0.1 |

Table h.i.iii: Number of Sensitizations: Median Regression for difference in change with time trend between groups.

| **Variable** | **Comparison** | **Effect** | **Std Error** | **lower CL** | **upper CL** | **p-value** |
| --- | --- | --- | --- | --- | --- | --- |
| **IA: Time*RD_Group** | 4 M vs. BL | -0.57 | 0.25 | -1.08 | -0.06 | 0.030 |
| **(HDM-SLIT vs. Placebo)** | 12 M vs. BL | -0.59 | 0.37 | -1.33 | 0.16 | 0.1 |
|  | 24 M vs. BL | -1.21 | 0.64 | -2.49 | 0.07 | 0.063 |

## ii. **In serum_Allergen extracts**

Table h.ii.i: Number of Sensitizations in serum.

| **Time** | **Group** | **Mean** | **SD** | **Median** | **Q1** | **Q3** | **Min** | **Max** | **N** |
| --- | --- | --- | --- | --- | --- | --- | --- | --- | --- |
| **Baseline** | Placebo | 2.07 | 1.16 | 2 | 1 | 2 | 1 | 5 | 15 |
| **Baseline** | HDM-SLIT | 1.83 | 1.04 | 1.5 | 1 | 2 | 1 | 4 | 18 |
| **4 Months** | Placebo | 2.27 | 1.28 | 2 | 1 | 3 | 1 | 5 | 15 |
| **4 Months** | HDM-SLIT | 1.72 | 1.18 | 1 | 1 | 2 | 1 | 5 | 18 |
| **12 Months** | Placebo | 2.64 | 1.78 | 2 | 1 | 3.75 | 1 | 6 | 14 |
| **12 Months** | HDM-SLIT | 2 | 1.41 | 1 | 1 | 2.75 | 1 | 5 | 18 |
| **24 Months** | Placebo | 3.15 | 2.08 | 3 | 1 | 5 | 1 | 6 | 13 |
| **24 Months** | HDM-SLIT | 1.75 | 0.93 | 1.5 | 1 | 2 | 1 | 4 | 16 |

Table h.ii.ii: Number of Sensitizations in serum: Median Regression for change with time trend.

| **Subgroup** | **Comparison** | **Effect** | **Std Error** | **lower CL** | **upper CL** | **p-value** |
| --- | --- | --- | --- | --- | --- | --- |
| **Within Placebo** | 4 M vs. BL | 0.21 | 0.22 | -0.24 | 0.66 | 0.3 |
|  | 12 M vs. BL | 0.46 | 0.33 | -0.20 | 1.13 | 0.1 |
|  | 24 M vs. BL | 1.06 | 0.52 | 0.00 | 2.11 | 0.049 |
|  | 12 M vs. 4 M | 0.23 | 0.32 | -0.41 | 0.87 | 0.4 |
|  | 24 M vs. 4 M | 0.86 | 0.45 | -0.05 | 1.77 | 0.062 |
|  | 24 M vs. 12 M | 0.49 | 0.34 | -0.19 | 1.17 | 0.1 |
| **Within HDM-SLIT** | 4 M vs. BL | -0.10 | 0.13 | -0.35 | 0.16 | 0.4 |
|  | 12 M vs. BL | 0.01 | 0.25 | -0.49 | 0.51 | 0.9 |
|  | 24 M vs. BL | 0.00 | 0.25 | -0.49 | 0.50 | 0.9 |
|  | 12 M vs. 4 M | 0.16 | 0.20 | -0.23 | 0.55 | 0.4 |
|  | 24 M vs. 4 M | 0.12 | 0.32 | -0.53 | 0.77 | 0.7 |
|  | 24 M vs. 12 M | -0.16 | 0.33 | -0.81 | 0.50 | 0.6 |

Table h.ii.iii: Number of Sensitizations in serum: Median Regression for difference in change with time trend between groups.

| **Variable** | **Comparison** | **Effect** | **Std Error** | **lower CL** | **upper CL** | **p-value** |
| --- | --- | --- | --- | --- | --- | --- |
| **IA: Time*RD_Group** | 4 M vs. BL | -0.31 | 0.23 | -0.77 | 0.14 | 0.1 |
| **(HDM-SLIT vs. Placebo)** | 12 M vs. BL | -0.40 | 0.43 | -1.27 | 0.46 | 0.3 |
|  | 24 M vs. BL | -1.05 | 0.61 | -2.27 | 0.18 | 0.092 |

## iii. **In skin_Allergen extracts**

Table h.iii.i: Skin: Number of Sensitizations.

| **Time** | **Group** | **Mean** | **SD** | **Median** | **Q1** | **Q3** | **Min** | **Max** | **N** |
| --- | --- | --- | --- | --- | --- | --- | --- | --- | --- |
| **Baseline** | Placebo | 1.4 | 0.74 | 1 | 1 | 2 | 0 | 3 | 15 |
| **Baseline** | HDM-SLIT | 1.22 | 0.55 | 1 | 1 | 1.75 | 0 | 2 | 18 |
| **4 Months** | Placebo | 1.71 | 0.99 | 2 | 1 | 2 | 0 | 4 | 14 |
| **4 Months** | HDM-SLIT | 1 | 0.49 | 1 | 1 | 1 | 0 | 2 | 18 |
| **12 Months** | Placebo | 1.79 | 1.58 | 1.5 | 0.25 | 3 | 0 | 4 | 14 |
| **12 Months** | HDM-SLIT | 1.33 | 1.08 | 1 | 1 | 1.75 | 0 | 4 | 18 |
| **24 Months** | Placebo | 2 | 1.54 | 1.5 | 1 | 2.5 | 0 | 5 | 12 |
| **24 Months** | HDM-SLIT | 1.67 | 1.35 | 1 | 1 | 2 | 1 | 6 | 15 |

Table h.iii.ii: Skin: Number of Sensitizations: Median Regression for change with time trend.

| **Subgroup** | **Comparison** | **Effect** | **Std Error** | **lower CL** | **upper CL** | **p-value** |
| --- | --- | --- | --- | --- | --- | --- |
| **Within Placebo** | 4 M vs. BL | 0.27 | 0.24 | -0.20 | 0.75 | 0.2 |
|  | 12 M vs. BL | 0.41 | 0.39 | -0.37 | 1.19 | 0.2 |
|  | 24 M vs. BL | 0.52 | 0.38 | -0.25 | 1.28 | 0.1 |
|  | 12 M vs. 4 M | 0.10 | 0.30 | -0.51 | 0.71 | 0.7 |
|  | 24 M vs. 4 M | 0.19 | 0.26 | -0.34 | 0.71 | 0.4 |
|  | 24 M vs. 12 M | 0.14 | 0.31 | -0.48 | 0.77 | 0.6 |
| **Within HDM-SLIT** | 4 M vs. BL | 0.00 | 0.17 | -0.34 | 0.35 | 0.9 |
|  | 12 M vs. BL | 0.03 | 0.34 | -0.66 | 0.71 | 0.9 |
|  | 24 M vs. BL | 0.29 | 0.30 | -0.32 | 0.89 | 0.3 |
|  | 12 M vs. 4 M | 0.27 | 0.28 | -0.28 | 0.83 | 0.3 |
|  | 24 M vs. 4 M | 0.56 | 0.32 | -0.09 | 1.20 | 0.089 |
|  | 24 M vs. 12 M | 0.18 | 0.36 | -0.54 | 0.91 | 0.6 |

Table h.iii.iii: Skin: Number of Sensitizations: Median Regression for difference in change with time trend between groups.

| **Variable** | **Comparison** | **Effect** | **Std Error** | **lower CL** | **upper CL** | **p-value** |
| --- | --- | --- | --- | --- | --- | --- |
| **IA: Time*RD_Group** | 4 M vs. BL | -0.51 | 0.24 | -0.99 | -0.02 | 0.040 |
| **(HDM-SLIT vs. Placebo)** | 12 M vs. BL | -0.31 | 0.44 | -1.19 | 0.56 | 0.4 |
|  | 24 M vs. BL | -0.22 | 0.46 | -1.14 | 0.71 | 0.6 |

## **i) Number of Sensitizations to Individual allergens in serum**

Table i.i.i: Number of sensitizations to allergen components

| **Time** | **Group** | **Mean** | **SD** | **Median** | **Q1** | **Q3** | **Min** | **Max** | **N** |
| --- | --- | --- | --- | --- | --- | --- | --- | --- | --- |
| **Baseline** | Placebo | 5.53 | 3.87 | 5 | 4 | 6 | 0 | 18 | 15 |
| **Baseline** | HDM-SLIT | 5.78 | 2.39 | 5 | 4 | 6.75 | 3 | 11 | 18 |
| **4 Months** | Placebo | 6.47 | 4.16 | 5 | 4.5 | 8.5 | 0 | 17 | 15 |
| **4 Months** | HDM-SLIT | 6.17 | 2.09 | 5 | 5 | 7.75 | 4 | 10 | 18 |
| **12 Months** | Placebo | 6.85 | 5 | 7 | 4 | 8 | 0 | 20 | 13 |
| **12 Months** | HDM-SLIT | 6.94 | 2.79 | 6 | 5 | 9 | 4 | 13 | 17 |
| **24 Months** | Placebo | 10.69 | 8.44 | 9 | 5 | 13 | 0 | 33 | 13 |
| **24 Months** | HDM-SLIT | 7.62 | 3.83 | 6 | 5.75 | 8 | 4 | 19 | 16 |

Table i.i.ii: Number of sensitizations to allergen components: Median Regression for time trend.

| **Subgroup** | **Comparison** | **Effect** | **Std Error** | **lower CL** | **upper CL** | **p-value** |
| --- | --- | --- | --- | --- | --- | --- |
| **Within Placebo** | 4 M vs. BL | 1.12 | 0.50 | 0.13 | 2.12 | 0.028 |
|  | 12 M vs. BL | 1.26 | 0.52 | 0.22 | 2.30 | 0.019 |
|  | 24 M vs. BL | 4.54 | 1.44 | 1.65 | 7.43 | 0.003 |
|  | 12 M vs. 4 M | 0.37 | 0.44 | -0.51 | 1.25 | 0.4 |
|  | 24 M vs. 4 M | 3.52 | 1.46 | 0.59 | 6.45 | 0.02 |
|  | 24 M vs. 12 M | 3.33 | 1.43 | 0.45 | 6.20 | 0.024 |
| **Within HDM-SLIT** | 4 M vs. BL | 0.71 | 0.25 | 0.22 | 1.20 | 0.006 |
|  | 12 M vs. BL | 1.00 | 0.47 | 0.06 | 1.93 | 0.038 |
|  | 24 M vs. BL | 1.62 | 0.81 | -0.01 | 3.26 | 0.052 |
|  | 12 M vs. 4 M | 0.60 | 0.52 | -0.44 | 1.65 | 0.2 |
|  | 24 M vs. 4 M | 1.23 | 0.81 | -0.39 | 2.85 | 0.1 |
|  | 24 M vs. 12 M | 0.39 | 0.63 | -0.86 | 1.65 | 0.5 |

Table i.i.iii: Number of sensitizations to allergen components: Median Regression for difference in time trend between groups.

| **Variable** | **Comparison** | **Effect** | **Std Error** | **lower CL** | **upper CL** | **p-value** |
| --- | --- | --- | --- | --- | --- | --- |
| **IA: Time*RD_Group** | 4 M vs. BL | -0.44 | 0.62 | -1.68 | 0.79 | 0.4 |
| **(HDM-SLIT vs. Placebo)** | 12 M vs. BL | -0.02 | 0.66 | -1.34 | 1.30 | 0.9 |
|  | 24 M vs. BL | -3.22 | 2.27 | -7.79 | 1.34 | 0.1 |

## j) **Inhibition-BAT (iBAT)**

## i. **Der p-extract**

Table j.i.i: Inhibition of basophil activation (%CD63+ basophils) in response to Der p-extract.

| **Time point** | **HDM-SLIT** | | | **Placebo** | | |
| --- | --- | --- | --- | --- | --- | --- |
|  | **Median** | **Q1** | **Q3** | **Median** | **Q1** | **Q3** |
| Baseline | 59.06 | 72.95 | 51.03 | 70.71 | 79.7 | 61.87 |
| 4 months | 36.54 | 51.07 | 28.36 | 73.61 | 78.87 | 51.84 |
| 12 months | 30.53 | 39.49 | 25.89 | 69.33 | 74.86 | 55.09 |
| 24 months | 29.86 | 40.25 | 24.61 | 60.66 | 71.52 | 56.12 |

Table j.i.ii: Dunn’s multiple comparison test within groups for change in inhibition of basophil activation by Der p-extract.

| **Comparisons** | **Rank sum diff.** | **p- Value** |
| --- | --- | --- |
| **HDM_SLIT** |  |  |
| 4 months vs BL | 6.00 | 0.18 |
| 12 months vs BL | 12.50 | 0.005 |
| 24 months vs BL | 13.50 | 0.0025 |
| 12 months vs 4 months | 6.50 | 0.15 |
| 24 months vs 4 months | 7.50 | 0.09 |
| 24 months vs 12 months | 1.00 | 0.8 |
| **Placebo** |  |  |
| 4 months vs BL | -1.00 | 0.8 |
| 12 months vs BL | 5.00 | 0.37 |
| 24 months vs BL | 1.00 | 0.26 |
| 12 months vs 4 months | -5.00 | 0.26 |
| 24 months vs 4 months | -4.00 | 0.18 |
| 24 months vs 12 months | 6.00 | 0.82 |

Table j.i.iii: Mann-Whitney U test for between-group differences of change in inhibition of basophil activation by Der p-extract.

| **Time point** | **p-value** | **Mean rank of HDM-SLIT** | **Mean rank of Placebo** | **Mean rank diff.** |
| --- | --- | --- | --- | --- |
| 4 months vs BL | 0.18 | 7.33 | 4.40 | 2.93 |
| 12 months vs BL | 0.004 | 8.50 | 3.00 | 5.50 |
| 24 months vs BL | 0.02 | 8.17 | 3.40 | 4.77 |

## ii. **Natural Der p 1**

Table j.ii.i: Inhibition of basophil activation (%CD63+ basophils) in response to nDer p 1.

| **Time point** | **HDM-SLIT** | | | **Placebo** | | |
| --- | --- | --- | --- | --- | --- | --- |
|  | **Median** | **Q1** | **Q3** | **Median** | **Q1** | **Q3** |
| Baseline | 61.66 | 73.59 | 54.88 | 58.19 | 69.09 | 49.09 |
| 4 months | 32.5 | 40.16 | 15.20 | 53.84 | 64.01 | 48.81 |
| 12 months | 14.04 | 39.04 | 4.96 | 53.94 | 60.78 | 46.57 |
| 24 months | 22.27 | 35.61 | 3.47 | 53.43 | 58.94 | 39.87 |

Table j.ii.ii: Dunn’s multiple comparison test within groups for change in inhibition of basophil activation by nDer p 1.

| **Comparisons** | **Rank sum diff.** | **p- Value** |
| --- | --- | --- |
| **HDM_SLIT** |  |  |
| 4 months vs BL | 7.50 | 0.06 |
| 12 months vs BL | 12.00 | 0.003 |
| 24 months vs BL | 10.50 | 0.01 |
| 12 months vs 4 months | 4.50 | 0.27 |
| 24 months vs 4 months | 3.00 | 0.46 |
| 24 months vs 12 months | -1.50 | 0.71 |
| **Placebo** |  |  |
| 4 months vs BL | 7.00 | 0.09 |
| 12 months vs BL | 4.00 | 0.32 |
| 24 months vs BL | 11.00 | 0.007 |
| 12 months vs 4 months | -3.00 | 0.46 |
| 24 months vs 4 months | 4.00 | 0.32 |
| 24 months vs 12 months | 7.00 | 0.09 |

Table j.ii.iii: Mann-Whitney U test for between group differences of change in inhibition of basophil activation by nDer p 1.

| **Time point** | **p-value** | **Mean rank of HDM-SLIT** | **Mean rank of Placebo** | **Mean rank diff.** |
| --- | --- | --- | --- | --- |
| 4 months vs BL | 0.002 | 9.50 | 3.507 | 6.00 |
| 12 months vs BL | 0.004 | 9.33 | 3.67 | 5.67 |
| 24 months vs BL | 0.026 | 8.83 | 4.17 | 4.67 |

## iii. **Natural Der p 2**

Table j.iii.i: Inhibition of basophil activation (%CD63+ basophils) in response to nDer p 2.

| **Time point** | **HDM-SLIT** | | | **Placebo** | | |
| --- | --- | --- | --- | --- | --- | --- |
|  | **Median** | **Q1** | **Q3** | **Median** | **Q1** | **Q3** |
| Baseline | 57.46 | 61.21 | 54.45 | 57.23 | 69.34 | 48.87 |
| 4 months | 41.1 | 54.88 | 33.05 | 57.38 | 63.71 | 43.16 |
| 12 months | 31.22 | 37.98 | 23.27 | 58.37 | 67.09 | 44.26 |
| 24 months | 32.53 | 36.59 | 25.58 | 51.48 | 65.63 | 42.59 |

Table j.iii.ii: Dunn’s multiple comparison test within groups for change in inhibition of basophil activation by nDer p 2.

| **Comparisons** | **Rank sum diff.** | **p- Value** |
| --- | --- | --- |
| **HDM_SLIT** |  |  |
| 4 months vs BL | 8.00 | 0.05 |
| 12 months vs BL | -11.00 | 0.007 |
| 24 months vs BL | -11.00 | 0.007 |
| 12 months vs 4 months | 3.00 | 0.46 |
| 24 months vs 4 months | -3.00 | 0.46 |
| 24 months vs 12 months | 0.00 | >0.9 |
| **Placebo** |  |  |
| 4 months vs BL | 1.00 | 0.80 |
| 12 months vs BL | 1.00 | 0.80 |
| 24 months vs BL | 4.00 | 0.32 |
| 12 months vs 4 months | 0.00 | >0.99 |
| 24 months vs 4 months | 3.00 | 0.46 |
| 24 months vs 12 months | 3.00 | 0.46 |

Table j.iii.iii: Mann-Whitney U test for between-group differences of change in inhibition of basophil activation by nDer p 2.

| **Time point** | **p-value** | **Mean rank of HDM-SLIT** | **Mean rank of Placebo** | **Mean rank diff.** |
| --- | --- | --- | --- | --- |
| 4 months vs BL | 0.04 | 8.67 | 4.33 | 4.34 |
| 12 months vs BL | 0.004 | 9.33 | 3.67 | 5.67 |
| 24 months vs BL | 0.002 | 9.50 | 3.507 | 6.007 |

**SuppInfo_Table 2. Occurrence of various symptoms.** Symptoms affecting the skin and airways were recorded at each visit. Data are presented as numbers and percentages (n, %).

| **Symptoms** | **Atopic dermatitis and pruritus** | | **Cough/wheezing** | | **Rhinitis** | | **Conjunctivitis** | |
| --- | --- | --- | --- | --- | --- | --- | --- | --- |
| **Time point** | **HDM-SLIT**  **n (%)** | **Placebo**  **n (%)** | **HDM-SLIT**  **n (%)** | **Placebo**  **n (%)** | **HDM-SLIT**  **n (%)** | **Placebo**  **n (%)** | **HDM-SLIT**  **n (%)** | **Placebo**  **n (%)** |
| **Baseline**  **(0 months)** | 1 (5) | 2 (13) | 12 (66) | 7 (47) | 7 (39) | 7 (47) | 6 (33) | 3 (20) |
| **4 months** | 1 (5) | 1 (6) | 7 (39) | 3 (20) | 8 (44) | 4 (27) | 6 (33) | 1 (7) |
| **12 months** | 0 | 2 (13) | 5 (28) | 3 (20) | 5 (28) | 4 (27) | 4 (22) | 1 (7) |
| **24 months** | 0 | 1 (6) | 6 (33) | 0 | 1 (5) | 5 (33) | 2 (11) | 1 (7) |

**SuppInfo_Table 3. Adverse events (AE).** i-ii: AEs are categorized as unrelated/unassignable to treatment and iii-iv: possibly related to treatment. Number of subjects experiencing AEs, type of AEs, and number of episodes for corresponding AE are presented as number and percentage of affected participants (n, %). Number of incidences for corresponding AEs type is presented in brackets (). † and ‡ indicate occurrence of multiple episodes per subject for the corresponding AE and abdominal pain, respectively.

|  | **HDM-SLIT (N=18)** | | **Placebo (N=15)** | |
| --- | --- | --- | --- | --- |
| **Type of adverse event (AE)** |  |  |  |  |
| **i. AEs unrelated/unassignable to treatment** | **n** | **%** | **n** | **%** |
| Any^†^ | 14 | 77.8 | 11 | 73 |
| Serious | 3 | 16.7 | 2 | 13 |
| Severe | 0 | 0.0 | 2 | 13 |
| Moderate | 13 | 72.2 | 10 | 67 |
| Mild | 11 | 61.1 | 9 | 60 |
| Multiple incidents | 11 | 61.1 | 7 | 47 |
| **ii. Types of AEs unrelated/unassignable to treatment and their incidences** |  |  |  |  |
| Gastrointestinal symptoms | 6 (6) | 33.3 | 4 (7) | 26.7 |
| Upper airway symptoms | 8 (20) | 44.4 | 8 (14) | 53.3 |
| Lower airway symptoms | 8 (17) | 44.4 | 7 (45) | 46.7 |
| Skin manifestations | 1 (1) | 5.60 | 1 (1) | 6.70 |
| Febrile/infectious episodes | 10 (21) | 55.6 | 9 (21) | 60.0 |
| Planned surgical interventions | 3 (3) | 16.7 | 1 (1) | 6.70 |
| Common paediatric diseases | 4 (5) | 22.2 | 4 (5) | 26.7 |
| Uncategorized events | 2 (2) | 11.1 | 2 (3) | 13.3 |
| **iii. AEs possibly related to treatment** |  |  |  |  |
| Any^†^ | 5 | 27.8 | 4 | 26.7 |
| Serious | 0 | 0.00 | 0 | 0.00 |
| Severe | 0 | 0.00 | 0 | 0.00 |
| Moderate | 1 | 5.60 | 2 | 13.3 |
| Moderate AE requiring medical care^‡^ | 0 | 0.00 | 1 | 6.70 |
| Moderate AE leading to permanent discontinuation of interventional product^‡^ | 0 | 0.00 | 1 | 6.70 |
| Moderate AE leading to temporary discontinuation of interventional product^‡^ | 1 | 5.60 | 0 | 0.0 |
| Mild | 4 | 22.2 | 2 | 13.3 |
| Experiencing multiple incidents | 2 | 11.1 | 0 | 0.00 |
| **iv. Type of AEs possibly related to treatment and their incidences** |  |  |  |  |
| Gastrointestinal symptoms | 2 (2) | 11.1 | 1 (1) | 6.70 |
| Upper airway symptoms | 2 (4) | 11.1 | 0 | 0.00 |
| Lower airway symptoms | 0 | 0.00 | 1 (1) | 6.70 |
| Skin manifestations | 0 | 0.00 | 2 (2) | 13.30 |
| Febrile-/ infectious-episodes | 0 | 0.00 | 0 | 0.00 |
| Planned surgical interventions | 0 | 0.00 | 0 | 0.00 |
| Common paediatric diseases | 0 | 0.00 | 0 | 0.00 |
| Uncategorized events | 2 (2) | 11.1 | 0 | 0.00 |
